# Merging Natural Biopolymers with Supramolecular Chemistry: Emulating the Native Extracellular Matrix’s Complexity

**DOI:** 10.1021/acsnano.5c10088

**Published:** 2025-08-15

**Authors:** Vera Sousa, Bruno Ladeira, Elisabeth Garanger, Sébastien Lecommandoux, E. W. Meijer, Patricia Y. W. Dankers, João F. Mano, João Borges

**Affiliations:** † CICECO − Aveiro Institute of Materials, Department of Chemistry, 56062 University of Aveiro, Campus Universitário de Santiago, 3810-193 Aveiro, Portugal; ‡ University of Bordeaux, CNRS, Bordeaux INP, LCPO, UMR 5629, F-33600 Pessac, France; § Institute for Complex Molecular Systems, Eindhoven University of Technology, 5600 MB Eindhoven, The Netherlands; ∥ Department of Biomedical Engineering, Laboratory of Chemical Biology, Eindhoven University of Technology, 5600 MB Eindhoven, The Netherlands; ⊥ Department of Chemical Engineering and Chemistry, Laboratory of Macromolecular and Organic Chemistry, Eindhoven University of Technology, 5600 MB Eindhoven, The Netherlands

## Abstract

The extracellular matrix (ECM) is one of the most striking natural self-assembled landscapes, essential for tissue integrity and cellular functions, where it orchestrates cell fate through a dynamic interplay of noncovalent interactions. Despite decades of research, there is still no scaffold that can replicate its nanostructural elegance and functional dynamic behavior. In this Perspective, we summarize cutting-edge approaches to reconstruct the ECM, putting an emphasis on either dynamic supramolecular designs or naturally sourced biopolymers. We then propose merging the natural with the synthetic world to enable hybrid cell-instructive materials that combine the dynamic mechanical profile, biomolecular composition and structural features of the ECM at all scales, from the nano- to the mesoscale, aiming to create a fully functional artificial ECM.

## Graphical abstract

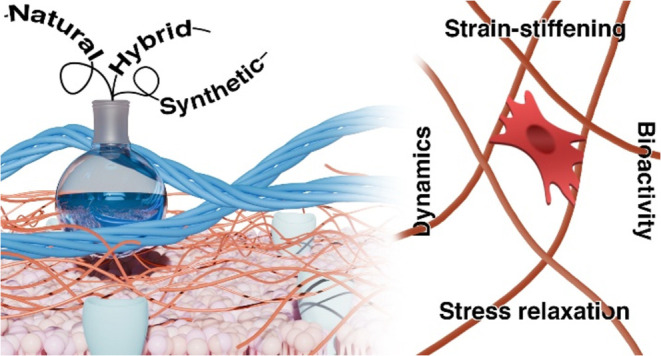

## Self-Assembly in Natural Systems

Self-assembly is a ubiquitous phenomenon in nature that showcases how life achieves nanostructural organization with minimal energy input. A superb yet simple example of effective control over the size of self-assembled biological nanostructures can be found in the tobacco mosaic virus (TMV). This plant pathogen efficiently builds its helical structure by assembling more than 2000 protein subunits around one molecule of viral RNA in a precise and controlled manner. In fact, the dynamic nature of this assembly mechanism enables self-replication, as TMV disassembles into individual protein subunits, and when reintroduced into physiological conditions, spontaneously reassembles into its original structure, forming an exact replica. Through a deep understanding of viral self-assembly mechanisms, it becomes feasible to rationally design intricate TMV-like nanoparticles that display heterogeneous surface ligands with spatial binding specificity,^1^ opening new avenues in bionanotechnology.

Another elegant example found in Nature, often overlooked due to its apparent simplicity, is the cell membrane formed through the supramolecular self-assembly of mainly lipids, proteins and carbohydrates. Such biological landscape demonstrates how minimalistic design, driven by the amphiphilic nature of lipids, can give rise to highly dynamic and functional structures.^2^ The spontaneous formation of phospholipid bilayers, driven by hydrophobic interactions at the nanoscale, not only establishes the basic compartmentalization required for life, but also assigns the unique stability to cell membranes and enables complex biological functions such as fusion, fission, and selective permeability. This subtle yet instructive self-organization process remains a guiding model for the design of synthetic nanosystems. Those include micelles, liposomes, and polymersomes which, despite acting as artificial cells,^3^ still fail to reach the full complexity of biological systems.

Few biological systems illustrate modular design as clearly as the cell cytoskeleton; a dynamic, self-assembling network whose components are designed to follow specific pathways.^4,5^ The building blocks of the cytoskeleton self-assemble to form dynamic and functional architectures with remarkable nanoscale precision and adaptability. These assembly processes are often controlled by enzymatic chemical reactions, making these natural systems the most beautiful examples of dissipative assemblies.^6^ Through the orchestrated assembly of filamentous structures—microtubules, actin filaments, and intermediate filaments—the cytoskeleton continuously builds and rebuilds the cell’s internal architecture in real time.^5^ This process facilitates physical and biochemical connections between the cell and its external environment, while also generating forces that enable cell movement and shape changes. However, these three main cytoskeletal polymers differ in their stiffness and dynamics, with microtubules being the stiffest and exhibiting the most complex assembly and disassembly dynamics. They switch, as a result of a chemical transformation, from growth to depolymerization and shrinkage, a process known as “dynamic instability”, which allows cells to rapidly reorganize their cytoskeleton whenever needed.^5^ Both actin filaments and microtubules are polarized polymers, featuring asymmetrical subunits that provide directional tracks for molecular motors. For instance, biological molecular machines associated with microtubules, including dyneins and kinesins, play essential roles in organizing the cytoskeletal networks, particularly in the formation of the mitotic spindle during cell division and the assembly of microtubule array during interphase.^7^ Similarly, myosins, which are associated with actin filaments in stress fibers, facilitate cell contraction by converting chemical energy into mechanical work, a process triggered by adenosine triphosphate (ATP) hydrolysis when ATP-bound myosin binds to actin filaments, generating a contractile force and movement within the actomyosin network.^8^ The fundamental role of the cytoskeleton in living cells has inspired the development of artificial cytoskeletons designed to mimic its mechanical and dynamic properties, serving as life-like artificial cell platforms.^9^

When we travel beyond the confines of the cell, we find the native extracellular matrix (ECM) as one of the most fascinating self-assembled structures. It is a complex matrix composed of cell-secreted molecules, including interwoven protein fibers, hydrophilic glycosaminoglycans (GAG) and proteoglycans, which assemble at the nanoscale through a wide variety of interactions, most of them noncovalent in nature. Collagen, a major structural protein of the native ECM, forms triple helices that self-assemble into fibrils and further spontaneously aggregate into larger fibers. Laminin organizes into polymeric, sheet-like basement membrane networks, and GAG, more specifically hyaluronic acid, generates highly hydrated, gel-like assemblies that fill the extracellular space.^10^ The intrinsic capacity of the ECM to self-assemble and self-organize in a dynamic manner is fundamental to many of its defining features. These include its ability for spatiotemporal controlled remodeling, responsiveness to biochemical and mechanical cues, and inherent capacity to provide structural support to cells and ensure the mechanical integrity of tissues, as well as guide tissue morphogenesis, differentiation and homeostasis. Therefore, it represents a prominent source of inspiration in the development of ECM-mimetic structures for cell culture. However, despite extensive research, we are still far from fully emulating the complexity, dynamic functional behavior, adaptive nature, and mechanical properties of this natural scaffold in life-like artificial matrices.

In this Perspective, we discuss past attempts to recreate the 3D architecture, biomechanical and nanostructural features of the ECM, focusing on the achievements of building blocks obtained from either natural sources or synthetic supramolecular polymers, as well as the pitfalls that have thus far prevented the scientific community from accomplishing this goal. Moreover, we highlight the recent progress in these fields and discuss the unrealized potential stemming from the synergistic interplay between the natural and synthetic worlds toward achieving functional artificial ECMs that better recreate living systems (Figure 1).

**Figure 1:**
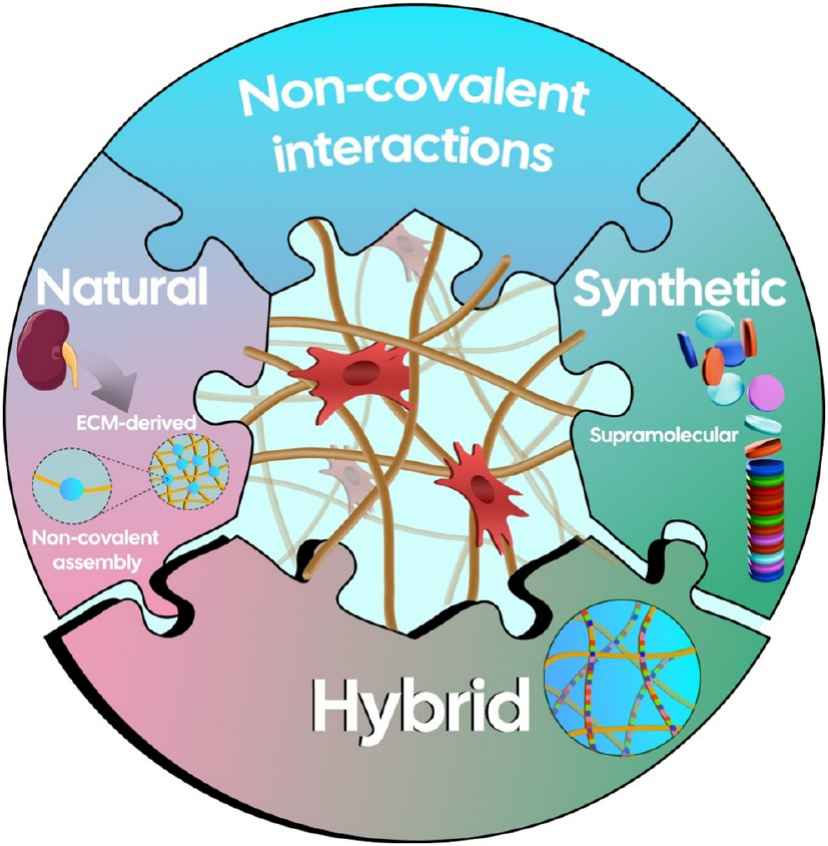
Merging the worlds of biology and chemistry: synergistic interplay between natural and synthetic (supramolecular) strategies for engineering hybrid materials.

## Challenge of Building a Biofunctional Artificial ECM

### Biochemical Composition of the ECM

From a biochemical standpoint, the ECM is a complex mixture, consisting of a wide variety of proteins, GAG and proteoglycans.^11–13^ The structural integrity of the ECM is maintained through a diverse group of proteins, including collagens, laminins, fibronectin or elastin (Figure 2). The most abundant components of the ECM are collagens, with collagen I being the most common example, which assemble to form a network of interwoven fibers that ensure the structural integrity of our tissues.^14^ Proteoglycans, on the other hand, consist of associations between a protein and negatively charged GAG chains, which can bind water molecules, and are thus responsible for the high water content of the ECM, its resistance to compressive forces, and the diffusion of small molecules. The structural features of the ECM are further reinforced by elastin, which imparts elasticity to the matrix and can be cross-linked alongside collagen by lysyl oxidases.^11^ Laminins and fibronectin bind to the resulting matrix while also promoting cell adhesion, thus connecting the cells to the surrounding fibrous network. These connections are mediated by adhesion motifs, small peptide sequences in these proteins that are recognized by cells and act as anchorage points, such as the tripeptide arginine-glycine-aspartic acid (RGD) motif. Not all proteins exert a structural role; in fact, while some contribute to specific tissue functions, others act as growth factors promoting cell proliferation, and some function as cell-secreted enzymes involved in matrix remodeling, either by introducing cross-links (*e.g.*, lysyl oxidase) or by digesting the matrix (*e.g.*, matrix metalloproteinases).

**Figure 2:**
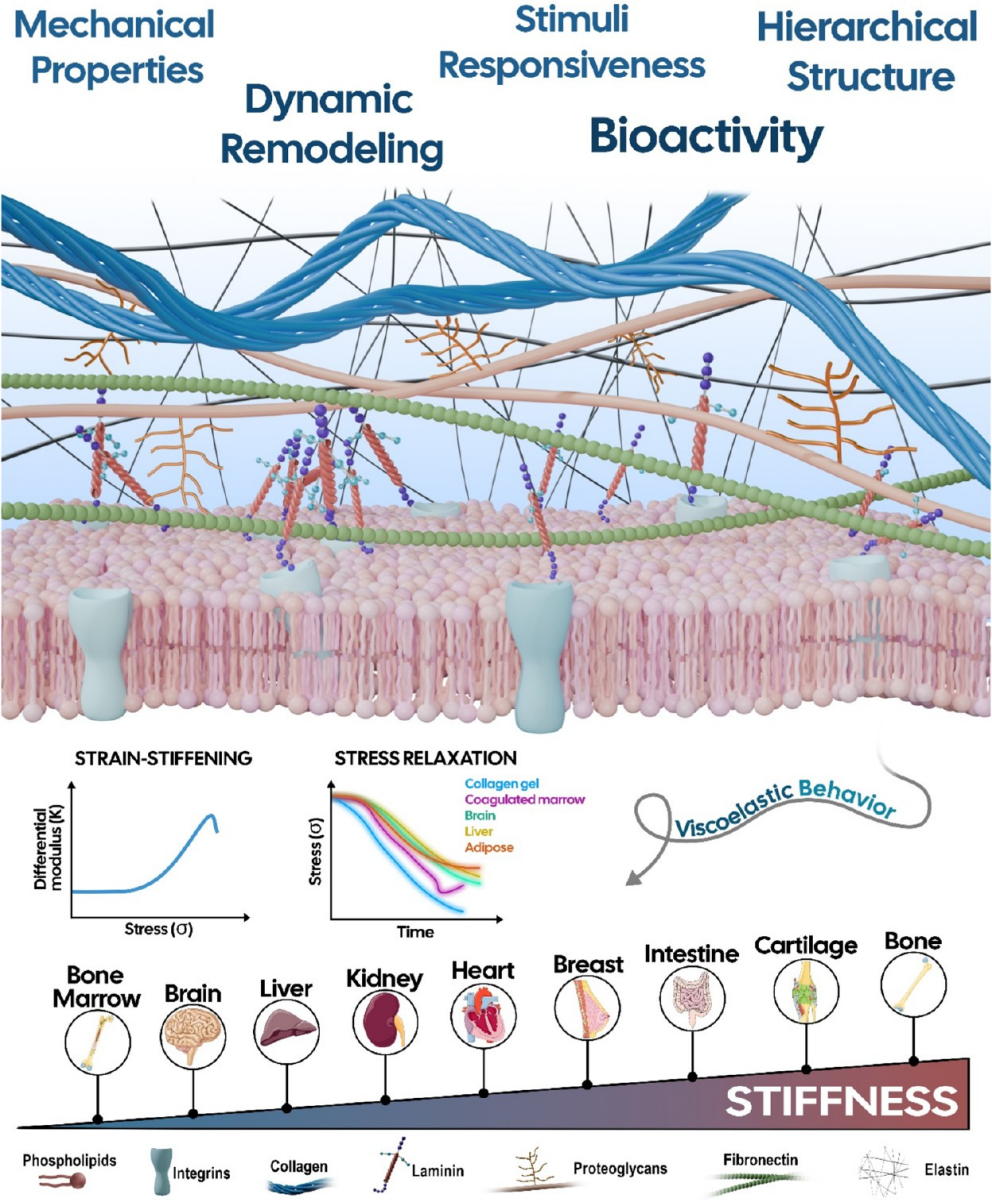
Schematic illustration of the ECM architecture highlighting major structural components and mechanical features typical of soft tissues, such as tissue-dependent stiffness, strain-stiffening behavior, and stress relaxation. Adapted with permission from ref 23. Copyright 2016 Springer Nature. Figure created using Blender software (4.3.2).

Owing to the progress in the field of proteomics and in technologies used to label ECM components, an ever greater assortment of these components has been identified, each playing a fundamental role in proper cell-matrix interactions, elucidating the challenges involved in producing an artificial ECM.^15,16^

### ECM Mechanics and Heterogeneity across Tissues: Rethinking the Universal Scaffold Paradigm

Beyond the biochemical complexity of the ECM, its biomechanical profile also exhibits unique features, which are tissue specific. For example, the stiffness of the ECM can range from 0.1 kPa in the neuronal or lung tissues up to 10 GPa in the bone (Figure 2).^17,18^ However, through extensive collaborative research efforts, many materials have already been developed that can match the stiffness displayed by various tissues.^19^

The most significant challenge to be addressed pertains to the fact that these human tissues are not static; instead, they exhibit highly dynamic properties and nanoscale features that play a fundamental role in tissue development, repair and function due to the noncovalent linkages involved in its assembly. These properties ensure that the ECM influences—and in turn is influenced by—cell activity. As cells pull on the matrix, they deform it, generating a strain to which the matrix can adapt to through its viscoelastic behavior. This allows the ECM to dissipate energy and release stress—a phenomenon known as stress relaxation—which has increasingly been recognized as a major factor determining cell proliferation, migration and differentiation.^20^ If the cells exert even greater forces on the matrix, the ECM will begin to stiffen in response. This nonlinear stress-stiffening response has been observed in many filamentous biopolymers, including collagen, which constitutes the ECM, as well as fibrin and the actin filaments that compose the cell cytoskeleton.^21^ These mechanical signals play a fundamental role in the communication between the cells and their surrounding ECM, and even in matrix-mediated cell–cell communication pathways.^22^ On top of the dynamics of noncovalent interactions, these adaptive properties are regulated by a manifold of biochemical covalent reactions.

The ability of cells to sense and transduce these mechanical cues from their environment—mechanotransduction—is fundamental to ensuring the stability of cell phenotypes in bioengineered tissues and, thus, these cues must be present in the scaffolds used to deliver them.

A closer inspection of the biochemical and biophysical profiles of the ECM reveals yet another challenge in building a biofunctional artificial ECM: the ECM varies from tissue-to-tissue. In fact, as we look across different tissues, we observe a wide variety of different ECMs that differ significantly in their composition and biomechanical properties.^24^ Moreover, as the ECM is inherently dynamic, its properties undergo constant remodeling over the course of an individual’s life, shaped by cellular activity, aging, or disease states, while also playing important roles in all of these processes.^11,24–27^ Thus, a single, universal artificial ECM does not exist; rather, it must be fine-tuned to replicate the properties of specific tissues.

## Natural-Based Scaffolds as ECM Mimics

Early attempts to replace damaged tissues relied simply on the transplantation of healthy tissues, whether sourced from the target tissues, or from more accessible tissues that are commonly discarded. From a materials design’s perspective, the need for complex processing or chemical modification of tissues is questionable, as these grafts promote regeneration through interactions between the intrinsic components of the tissue graft and the cells in the targeted organs. As the supply of tissues is limited, the challenge of engineering tissues *in vitro* has emerged, although several questions remain unanswered. How can we generate sufficient tissues to meet the needs of the population? Where can the building blocks for these tissues be sourced from? And how can we design the tissue grafts to meet the characteristics of the targeted tissues and promote their regeneration? The latter question has stimulated the development of hydrogel-based systems, as these were thought to better recreate the dynamic 3D architecture, high water content, porosity, and mechanical properties of human tissues, enabling cell culture in physiological conditions.^28^ However, the use of hydrogels alone cannot fully replicate the ECM, as their properties are heavily influenced by the materials used to create them, which can either align with or diverge from the ECM’s characteristics. The most suitable materials for this purpose are those that can better mimic or guide the nanostructural organization, the dynamic 3D architecture, and the functional properties of the ECM. In this context, materials sourced from Nature present considerable advantages as they innately share multiple similarities with the ECM. These considerations have led to the development of chemical and engineering approaches to identify, produce and process natural macromolecules and matrices that can be used as building blocks for enabling new tissues. In this section, we will focus on these macromolecules and the approaches used to build tissues from them.

### The Golden Standard of ECM-Mimetic Materials

The most popular option to produce ECM-mimetic environments consists of commercialized ECM extracts derived from the basement membrane of Engelbreth-Holm-Swarm (EHS) mouse sarcoma, such as Geltrex, Cultrex and Matrigel. Matrigel, in particular, is still widely considered the golden standard *in vitro* model for numerous applications, including organoid culture, disease models and pharmacological testing. Owing to its composition, richness in growth factors and common basement membrane components such as collagen IV, entactin, perlecan, and multiple isoforms of laminin, Matrigel promotes the adhesion and bioactivity of a wide variety of cell lines.^29,30^ However, it has increasingly been recognized that EHS matrix-based platforms exhibit significant limitations, which hamper their use in clinical applications.

First, the composition of these matrices remains ill-defined, with many low-abundance components potentially remaining undiscovered. Additionally, the presence and concentration of these components exhibit considerable variability, which, in turn, produces variability in the mechanical properties of the subsequent hydrogels, which can differ significantly between batches and even within different regions of the same scaffold.^31^ This heterogeneity greatly limits the reproducibility of findings obtained using these scaffolds. Aside from this variability, the lackluster mechanical properties of EHS matrices produce additional obstacles. For example, the inherent softness of this matrix impairs the handling of the hydrogels and threatens their mechanical stability. Additionally, it also fails to reproduce the biomechanical profile of the native tissues as it does not exhibit certain properties of the native ECM that are essential for proper cell-matrix interactions, such as strain-stiffening behavior.^32^

Finally, the source of these matrices also introduces numerous concerns. As these matrices are of animal origin, there is potential for the transmission of zoonotic pathogens, as well as an increased possibility of rejection due to its xenogeneic character, limiting their application *in vivo.* ^33^ These issues are further compounded by their extraction from tumor tissue, which raises safety concerns, while also signifying that these matrices lack tissue-specific components that may be essential in reproducing the properties of each individual tissue.^24,33^

These drawbacks have fueled the search for alternatives to EHS matrices, both from natural and synthetic origins. In this section, we will focus on the candidates that are sourced directly from Nature.

### Recreating the ECM, One Component at a Time

Among the potential candidates to replace EHS matrices, the simplest option is to resort to individual components of the ECM, such as collagen or fibrinogen.^34^ In particular, collagen I exhibits great promise as it is the most abundant component of the ECM, and it can be isolated from a wide variety of tissues, ensuring its broad availability. As such, it has become one of the most extensively studied materials in tissue engineering, following attempts to identify strategies that can be used to tailor collagen-based matrices toward different applications.^35^

While scaffolds generated from native collagen have resulted in significant achievements in the field of tissue engineering, these accomplishments are further compounded by the extensive literature surrounding its derivatives, most prominently, gelatin. Gelatin is generated by applying a thermal treatment to collagens, accompanied by hydrolytic denaturation, which can be performed in acidic or alkaline condition, generating two forms of gelatin with different properties and behaviors.^36^ Although gelatin loses much of the architecture and structural features of collagen in this process, it retains its biochemical composition, while exhibiting vastly improved solubility, reduced immunogenicity and significantly lower costs.^37,38^ However, without additional modifications, gelatin is unsuitable for tissue engineering applications as it exhibits a thermoreversible gelation mechanism, generating hydrogels at low temperatures and solubilizing at physiological temperature. For this reason, gelatin is routinely subjected to chemical modification to improve its long-term integrity.

The most common strategy used to accomplish this goal pertains to the introduction of photopolymerizable methacryloyl moieties, generating gelatin methacryloyl, most commonly known as GelMA.^38^ GelMA generates hydrogels with vastly improved mechanical properties when compared to gelatin, native collagen or EHS-derived matrices. Additionally, this chemical approach grants the ability to fine-tune these properties by adjusting the degree of substitution, the concentration of the polymer, the irradiation process, or even through mechanical stimulation.^38–40^ Due to the accomplishments of GelMA-based platforms, this strategy has since been popularized and translated into other materials, including other ECM components, such as the GAG hyaluronic acid (HA) and chondroitin sulfate.^41^

While these biopolymers present greater biosimilarity to native tissues, they remain single components of the ECM and, therefore, they cannot fully represent the inherent complexity and multicomponent nature of the ECM. While mixing multiple of these components can provide greater similarity to native tissues, as we will explore in later sections, the most accessible way to achieve complex matrices is by exploiting resources that already exhibit this same complexity.

### Decellularization—A Path to Recycle Discarded Tissue

A well-established strategy to recreate the ECM is to repurpose previously existing ECM by harvesting it from tissues or organs, thus retaining the biochemical and physiological cues that make the ECM so unique. This process is then followed by removing native cells and repopulating the matrix with other cells. Animal-based tissues, usually of porcine or bovine origin, are used as the source for this decellularized extracellular matrix (dECM).^24,42^ However, the animal origin of these materials poses serious ethical and safety concerns. Additionally, the architecture and composition of these animal-derived tissues may also not be able to reproduce the properties of human tissues.

For this reason, human tissues have been increasingly explored as sources for xeno-free ECM, through the development of procedures that allow the decellularization of multiple human tissues and even whole organs.^43^ However, the availability of most human tissues is much more limited than those of animal origin, as they can only be obtained from deceased donors, preventing the large-scale application of human dECM-based approaches. This has encouraged research into tissues that exhibit greater availability, such as those that are commonly discarded or those that can be safely harvested from living donors, including the omentum,^44^ adipose tissues,^45^ blood,^46^ or perinatal tissues,^47^ which encompass the placenta, fetal membranes and the umbilical cord (Figure 3A).

**Figure 3:**
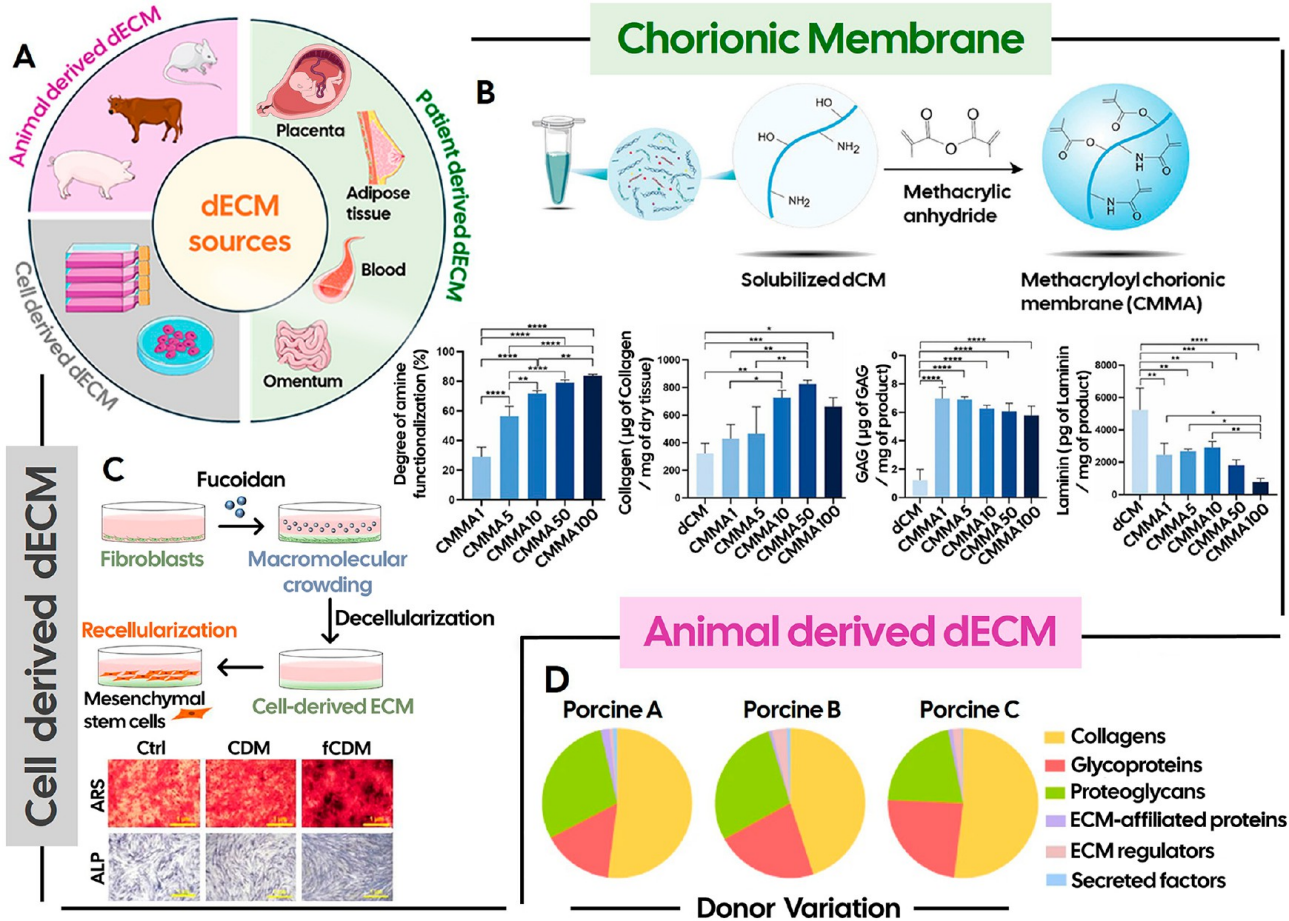
(A) Sources of decellularized extracellular matrix (dECM), including animal-derived, patient-derived, and cell-derived origins; (B) Chemical route for the synthesis of methacrylated chorionic membrane (CMMA) from decellularized chorionic membrane (dCM) (top), and effect of the modification degree on amine functionalization, collagen, glycosaminoglycans, and laminin content (bottom). Different degrees of modification (CMMA1, CMMA5, CMMA10, CMMA50, and CMMA100) were tested by increasing the amount of methacrylic anhydride used in the chemical reaction, as shown by the higher functionalization of amines (ranging from 29 to 84% for CMMA1 and CMMA100, respectively). Collagen content increased with higher modification degrees, while GAG levels were generally higher in all CMMA samples when compared to dCM. Laminin content showed a significant decrease upon modification. Reproduced with permission from ref 49. Copyright 2025 John Wiley and Sons; (C) Fucoidan, applied as a macromolecular crowder, enhanced the human dermal fibroblast-derived ECM (CDM) production and deposition (top). The resulting fucoidan-induced cell-derived matrix (fCDM) promoted the osteogenic differentiation of mesenchymal stem cells, as demonstrated by an alkaline phosphatase (ALP) and alizarin red s (ARS) staining assay (bottom). Scale bars are 1 μm. Reproduced with permission from ref 58. Copyright 2022 Elsevier; (D) Batch-to-batch variability in decellularized intestine-derived ECM of three different porcine donors (A–C). While the overall profiles of matrisome proteins showed little variation between donors, more pronounced differences, particularly in glycoproteins and proteoglycans, were observed between donors A and C. Reproduced with permission from ref 33. Available under a CC-BY 4.0. Copyright 2022 Springer Nature.

Previous research from one of our groups has successfully translated the same chemical approach used to generate GelMA into some of these human tissues, producing photopolymerizable biomaterials from human amniotic membrane,^48^ human chorionic membrane (Figure 3B),^49^ and human platelet lysate (hPL).^50^ In particular, hPL represents a unique resource to bestow biochemical functionality to scaffolds. Although it lacks the architecture of the ECM and many of its main components, including collagen I and GAG, it contains a wide variety of growth factors that promote cell adhesion and proliferation, wound healing, ECM deposition and angiogenesis.^46^ These components are essential to fully recreate the native ECM, which also presents cells with a diverse cocktail of soluble factors that assist in guiding cell fate and tissue maturation. However, they are absent from other Nature-derived materials, including dECM-derived matrices, as many of the native factors in the ECM are lost during the decellularization process.^51^ For this reason, hPL has been increasingly incorporated into both natural and synthetic scaffolds to enhance their biochemical functionality.^52,53^

Nevertheless, harvesting ECM from human tissues limits the pool of available tissues from which the ECM can be extracted. While it has been reported that certain dECM-based scaffolds have demonstrated the potential to regenerate tissues beyond their tissues of origin,^54,55^ another possibility to overcome the limited supply of most human tissues is to resort to cell-derived ECM (Figure 3C). By culturing cells from specific cell types and harvesting the ECM they secrete, it is possible to generate ECM tailored to specific cell types and tissues, and even emulate the properties of diseased tissue.^56,57^ In addition, biochemical and biomechanical stimuli can be used to condition the cells and generate ECM with improved properties, such as increased stiffness, immunomodulatory capabilities or the ability to guide cell differentiation.^58–60^ The main limitation of these materials pertains to their supply, as upscaling the production of cell-derived ECM to meet the requirements for large-scale clinical application remains a major obstacle. However, it is expected that this goal will become more and more attainable over time, following the evolution of technologies used for large-scale cell production, which have achieved great progress in recent years.^61^ By using large-scale cell expansion techniques, it may also be possible to generate vast quantities of matrix from a single cell line, standardizing the production process and reducing differences between ECM batches.

The selection of a suitable source for dECM must consider several factors, including availability, immunogenicity, and compositional relevance. Animal-derived ECMs are generally more available but may differ from human tissues in critical ways. Human-derived matrices offer lower immunogenicity, particularly when autologous, but are limited by donor availability. Cell-derived ECMs provide greater compositional versatility, though they can be more technically demanding to produce. However, many challenges still hinder the application of these scaffolds, regardless of source. Batch-to-batch variability, either across tissue samples from the same donor or between different donors (Figure 3D), remains a considerable hurdle in the development of dECM-based approaches. Although batch-to-batch variability can be partially mitigated by enriching or depleting the matrix from components that may influence cell response,^62–64^ this strategy fails to fully eliminate the issue.^65^ Additionally, while dECM-derived materials can impart a wide diversity of biochemical cues to cells, this complexity acts as a double-edged sword, obscuring many of the underlying mechanisms guiding cell fate. The use of decellularized ECM also raises concerns regarding residual immunogenic components.^66^ The ability of dECM-based scaffolds to replicate the characteristics of native tissues is also constrained by the decellularization process, which inevitably induces deleterious changes to the matrix. These changes may include disruption of the 3D ultrastructure and nanostructural features, alterations in mechanical properties, and loss of key ECM components, particularly soluble factors that are essential to ensure adequate cell behavior, as previously discussed.^51,66^ Another limitation of all dECM-based platforms, whether cell or tissue derived, whether of animal or human origin, is that much like EHS-derived matrices, their gelation capacity is limited and slow. Moreover, the mechanical properties of the resulting scaffolds exhibit a lower bound (defined by the minimum required concentration to generate a gel), and a low upper bound (dictated by the reduced solubility of dECM), compromising the mechanical integrity of the scaffolds by generating excessively soft matrices that fail to reproduce the stiffness of many tissues.^67–69^

These issues have been addressed through the chemical modification of these matrices with functional moieties that can be cross-linked, generating semisynthetic materials with reduced gelation times that provide a greater degree of control over their mechanical properties, potentially mitigating the issue of batch-to-batch variability.^47^ Throughout this section, we have extensively discussed the possibility of introducing photopolymerizable methacryloyl moieties into natural materials, which has become a prevailing approach in tissue engineering.^38,41,48–50^ Light-based click chemistry approaches can be applied,^70^ as well as other click chemistry mechanisms that do not require light,^71,72^ thereby significantly diversifying the available chemical toolkit to generate improved scaffolds from natural macromolecules and dECM. Other cross-linking strategies may include the use of genipin, glutaraldehyde or carbodiimide coupling chemistries,^73^ or even enzyme activity.^74^

However, many of the most popular chemical strategies to produce cross-links in these materials rely on the formation of covalent bonds, leading to static matrices with atypical biomechanical profiles, straying from the biophysical characteristics of the ECM. As mentioned previously, while covalent cross-links are present in our tissues due to the activity of enzymes such as lysyl oxidases, the assembly of the ECM mostly relies on supramolecular interactions between its constituent macromolecules. Thus, it has been recognized that, to fully reconstruct the ECM, the contributions of these noncovalent assembly mechanisms must not be overlooked. In the following section, we explore the abundance of noncovalent interactions present in natural systems, and how they have been leveraged to overcome the limitations of Nature-derived materials and generate scaffolds with biosimilar mechanical properties. Harnessing the design of wholly natural noncovalent materials unveils novel opportunities to emulate the native ECM by combining bioactive constituents of the ECM itself or structurally similar components, with the dynamic nature of supramolecular interactions. These specific interactions will be discussed in the next section.

## Avenues toward the Noncovalent Assembly of Natural Biopolymers

### Metal-Ion Coordination

The formation of coordination complexes with metal ions is a widespread biochemical strategy used to maintain homeostasis and regulate tissue function. Porphyrin-derived molecules, such as the *heme* group in hemoglobin or the chlorophylls present in plant cells, require coordination with metal ions to enable their roles in oxygen uptake and photosynthesis, respectively.^75^ However, many other bio­(macro)­molecules, such as polysaccharides, proteins, and nucleotides, also exhibit the ability to coordinate with metal ions,^76^ a property that can serve as a basis for assembling artificial matrices using metal ions as cross-linkers.

One prominent example in tissue engineering applications is the calcium-dependent gelation of alginate (ALG), which generates soft matrices that emulate the mechanical properties of living tissues. ALG is a polysaccharide derived from brown algae, composed of (1,4) β-d-mannuronic acid (M) and α-l-guluronic acid (G) residues, which is cross-linked in the presence of divalent cations, such as Ca^2+^, Sr^2+^, Ba^2+^, Mn^2+^, Cu^2+^, or Zn^2+^.^77,78^ The interactions between the ALG chains and these cations are highly dependent on both the selected cation and the quantity/distribution of M and G units, providing ample opportunities to fine-tune the mechanical properties of ALG-based hydrogels.^77–79^ Moreover, other factors such as metal-ion concentration, ALG molecular weight, and chemical modification of the polymer with spacer groups also influence the biomechanical properties of the hydrogels. These factors provide avenues to modulate the stiffness and the stress relaxation behavior in an independent manner, thereby providing insights into how they individually affect the behavior of cells and cell-based structures, as well as tissue morphogenesis.^23,80–82^

Another widely used polysaccharide for enabling hydrogel formation is chitosan (CHT), obtained through the deacetylation of chitin, the second most abundant biopolymer in Nature after cellulose.^83^ CHT has been increasingly used in biomedical applications due to its biocompatibility, biodegradability, noncytotoxicity and antibacterial activity.^84,85^ CHT is also able to establish interactions with metal cations.^86,87^ However, as a polycation, it can also coordinate with anionic species, such as citric acid^88^ or phosphate-containing molecules like glycerylphytate.^89^ Interactions between CHT and the polyanionic molecule tripolyphosphate have been exploited to form hydrogels,^90^ membranes,^91^ and nanoparticles,^92^ or even to stiffen already existing hydrogel networks.^93^ The modification of CHT with tricine—a noncanonical amino acid bearing three hydroxyl groups—can also promote hydrogen bonding, which significantly strengthens the hydrogel network, resulting in an extrudable material.^94^ A major limitation of CHT pertains to its insolubility at physiological pH, thus being unsuitable for cell encapsulation. However, this drawback can be addressed through chemical modification of CHT to produce derivatives that are soluble at physiological pH, such as CHT lactate,^89^ or quaternized CHT.^95^ Another possibility is the addition of β-glycerophosphate, a known inductor of osteogenesis, which interacts with CHT, enabling it to remain in solution at neutral pH.^96,97^ Upon heating to physiological temperature, the CHT-β-glycerophosphate mixture undergoes gelation,^98^ reproducing the thermosensitive behavior of the ECM.

Beyond polysaccharides, many protein motifs also exhibit the ability to bind metal ions. For instance, the zinc finger domain, which coordinates Zn^2+^ ions, is an important component of many proteins that function as a transcription factor.^99^ In fact, metal ions are an important coadjuvant in many enzymes, assisting in the regulation of the enzyme activity,^100^ while also playing important roles in proteins associated with surface adhesion, electron transfer or signal transduction.^101^ Amino acids bearing hydroxyl or carboxyl groups may bind metal ions,^101^ and so can phenylalanine residues.^102^ However, metal ion binding in proteins is primarily mediated by cysteine,^102,103^ tryptophan,^104^ and histidine.^102^ Histidine residues, in particular, exhibit the ability to coordinate with a wide variety of ions, providing a considerable degree of control over the stiffness and the stress relaxation properties of the resulting scaffolds.^105^ The resulting properties of the scaffolds vary depending on the chosen cation,^106^ as well as its redox state,^107^ providing ample opportunities to fine-tune the mechanical properties of the hydrogels. The coordination with divalent cations has also been explored to expand the limited stiffness range of protein-based materials (i.e., bovine serum albumin) by inducing reversible stiffening of the matrix, while also granting it shape memory properties (Figure 4A).^68^ In addition, the metal-binding properties of these proteins can be harnessed for ion delivery to guide cell differentiation and tissue formation.^103^

**Figure 4:**
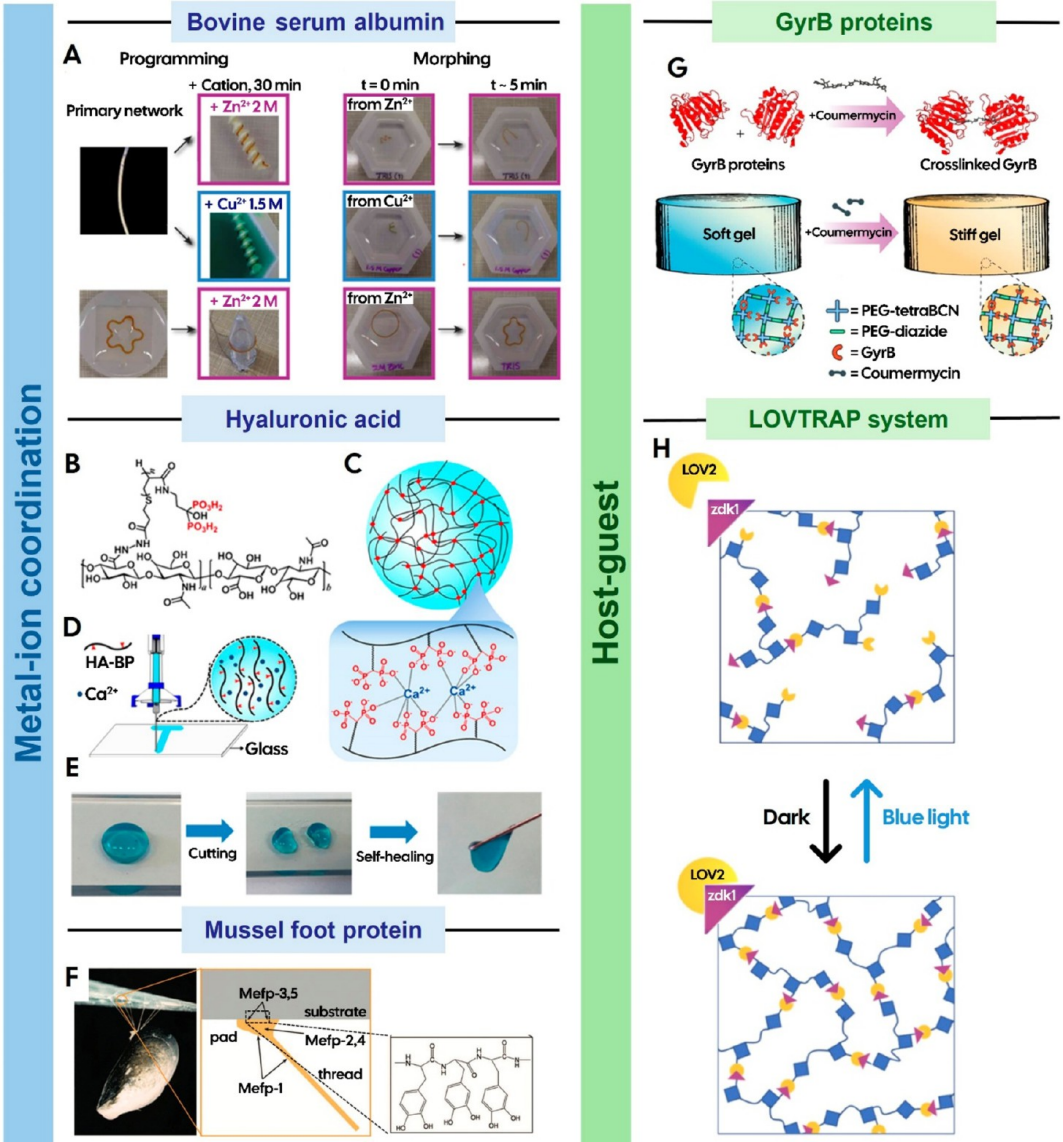
(A) Shape-memory behavior in protein-based hydrogels: Bovine serum albumin (BSA)-based hydrogels were synthesized via a photoinitiated reaction using ammonium persulfate and tris­(bipyridine)­ruthenium­(II) chloride. Initially molded into cylindrical shapes, the preformed hydrogels were reprogrammed into spring-like structures by immersion in 2 M Zn^2+^ and 1.5 M Cu^2+^ solutions for 30 min, using a negative mold (top, left). Similarly, BSA hydrogels cast in a flower shape using a silicone mold were reconfigured into ring shapes after immersion in a Zn^2+^ solution (bottom, left), demonstrating tunable shape-memory capabilities in response to metal-ion coordination. Hydrogels programmed into a specific shape can retain their conformation after immersion in certain ionic solutions. However, upon exposure to a Tris buffer, the material rapidly returns to its original shape, illustrating a reversible shape-memory behavior (right). Reproduced with permission from ref 68. Distributed under a CC-BY-NC 4.0. Copyright 2020 The Authors, some rights reserved; exclusive licensee The American Association for the Advancement of Science; (B) Bisphosphonate-modified hyaluronic acid interacts with Ca^2+^ ions (C) to generate a printable hydrogel that displays shear-thinning (D) and self-healing behavior (E). Reproduced with permission from ref 108. Copyright 2017 American Chemical Society; (F) Image of the mussel*Mytilus edulis*adhered to a glass surface, showing the byssal threads and adhesive pads (left). The foot proteins of*M. edulis*­(Mefps) exhibit distinct distribution patterns, with Mefp-3 and Mefp-5 localized at the pad–substrate interface, both of which possess the highest DOPA contents among Mefps (right). Reproduced with permission from ref 109. Copyright 2006 National Academy of Sciences, U.S.A.; (G) Coumermycin-induced dimerization of DNA gyrase subunit B (GyrB) as a cross-linking mechanism to stiffen hydrogels. Reproduced with permission from ref 110. Available under a CC-BY 3.0. Copyright 2023 The Royal Society of Chemistry; (H) LOVTRAP system—Zdk1 exhibits high affinity binding to LOV2 in the dark and a reduced affinity under blue light exposure. Reproduced with permission from ref 111. Copyright 2021 American Chemical Society.

Ionic gelation can also be introduced in biopolymers that do not inherently exhibit this behavior by functionalizing them with metal-coordinating moieties. For example, HA modified with biphosphonate groups (Figure 4B) can coordinate with Ca^2+^ ions (Figure 4C) to form a printable material with shear-thinning (Figure 4D) and self-healing properties (Figure 4E).^108^

Compounds bearing phenol groups, such as catechols, also exhibit the ability to coordinate with metal ions, generating strong noncovalent interactions. A notable example in Nature is mussel foot protein 1 (Figure 4F), which contains the noncanonical amino acid 3,4-hydroxyphenylalanine (hereinafter referred to DOPA). This amino acid is capable of coordinating with iron ions to form strong noncovalent interactions that allow mussels to adhere to a wide variety of organic and inorganic surfaces under both wet and dry conditions.^109,112^ These catechol groups, and other phenolic compounds, can coordinate with a broad range of metal ions to create highly dynamic, reversible cross-links that enhance hydrogel stiffness and toughness,^113,114^ while also providing control over matrix viscoelasticity.^115^ Some catechol groups, such as DOPA, are highly susceptible to oxidation, generating strong covalent bonds that improve the stability and toughness of the matrix, while others, such as hydroxypyridinone (HOPO), are more resistant to oxidation, allowing them to maintain the dynamic properties of the matrix in the long-term.^116,117^ Combining the capabilities of these two groups appears to promote the differentiation of mesenchymal stem cells into osteoblasts and monocytes into osteoclasts, thereby mimicking the cellular microenvironment of bone tissue without the need for biochemical inductors of osteogenesis.^118^

### Host–Guest Interactions

Beyond metal-ion coordination, many other interactions in Nature rely on molecular recognition. A well-known example is the binding between an enzyme and its substrates or cofactors, which follows the classic “lock and key” principle. The binding of a ligand triggers changes in protein conformation, modulating the stability, mechanical properties, and even the macroscopic structure of protein-based hydrogels.^119,120^ This phenomenon has been most prominently demonstrated in systems utilizing calmodulin, a naturally occurring protein that selectively binds to many target proteins and other small molecules in the presence of Ca^2+^, thereby regulating their function.^121^ Upon binding to Ca^2+^ ions, calmodulin adopts a dumbbell-like extended conformation. However, when it binds to its other ligands, it collapses, which translates into macroscopic volume changes in the hydrogels incorporating this protein, contributing to their dynamic character and providing control over the diffusion of small molecules.^122,123^

In addition, these selective noncovalent interactions between proteins and their ligands offer powerful avenues for designing novel, Nature-inspired assemblies with dynamic and tunable properties. In this regard, the DNA gyrase subunit B (GyrB) is able to bind to two different small antibiotics, coumermycin and novobiocin, with similar affinity. However, coumermycin can bridge two separate GyrB molecules, while novobiocin binds only one. This has prompted the use of coumermycin-induced dimerization of GyrB molecules as a cross-linking mechanism to form or stiffen hydrogels (Figure 4G).^110,124^ Through the competitive binding of novobiocin, it is possible to reverse the stiffening in a controlled manner, or even prompt the dissolution of the hydrogels altogether, potentially aiding in cell or drug delivery applications.

Harnessing protein dimerization and protein oligomerization has become an emerging approach to promote the noncovalent assembly of proteins and protein-based materials.^125^ In some cases, this approach can be driven by the interaction between specific protein motifs, leading to the supramolecular polymerization of protein fragments, a process known as protein fragment complementation or protein fragment reconstitution.^126,127^ In other cases, this mechanism is mediated by small molecule ligands that are recognized by two protein motifs, a process known as chemically induced dimerization (CID).^128^ This strategy takes inspiration from natural mechanisms, such as the rapamycin-induced heterodimerization of FK506 binding protein (FKBP) and FKBP12-rapamycin binding protein (FRB), which regulates the intracellular localization of proteins of interest fused to the FRB.^129,130^

Protein oligomerization may also be dependent on external stimuli, offering a route to control the stiffening and softening of the scaffold postfabrication, and triggering its disaggregation once it has fulfilled its role. This concept has been most prominently explored in proteins that undergo oligomerization as a response to light, which mimic the behavior of synthetic photoswitchable systems, such as those dependent on interactions between azobenzene and cyclodextrins/cucurbiturils. For example, the cyanobacterial phytochrome 1 (Cph1) forms dimers upon exposure to red light (∼660 nm), which dissociate when irradiated with far-red light (∼740 nm), leading to changes in the stiffness and stress relaxation properties of the resulting hydrogels.^131^ In fact, there are multiple photoswitchable protein domains that respond to different wavelengths, including UVR8,^132^ Dronpa145N,^133^ the LOVTRAP system (Figure 4H),^111^ or the C-terminal adenosylcobalamin binding domain of photoreceptor proteins (CarHc).^134^

By combining proteins responsive to different wavelengths within the same system, it is possible to achieve precise control over material properties, while also facilitating modular tissue engineering approaches by granting greater control over the spatiotemporal properties of the scaffolds. For instance, the presentation of photoswitchable proteins on the outer surface of cell membranes has been reported.^135,136^ As the cells were modified with two separate proteins that respond to different wavelengths, it was possible to achieve precise spatiotemporal control over the distribution and adhesion of cells.

Another alternative is to leverage molecules that naturally form host–guest interactions with many different molecules, including biologically relevant ones. Examples of such molecules are cucurbiturils and cyclodextrins, both of which can form inclusion complexes with bulky hydrophobic moieties, such as adamantane. These interactions can be exploited to create noncovalent networks. For instance, cyclodextrins and adamantane moieties have been incorporated into polymer chains to develop hydrogels and supporting baths for bioprinting with extensive self-healing abilities.^137,138^

Another possibility is to modify the hydroxyl groups of cyclodextrins with a polymerizable molecule to convert the cyclodextrin into a supramolecular cross-linking agent. An example of the latter is the modification of cyclodextrins with acryloyl groups, which can be cross-linked in the presence of UV light. By allowing the modified cyclodextrin to interact with polymeric chains bearing hydrophobic side groups, it effectively decorates the polymer with acryloyl moieties that can undergo photopolymerization, forming small nanoclusters of cyclodextrins that bind different polymeric chains together.^139^ By changing the hydrophobic moieties used to generate this binding, it is possible to modulate the dynamics of the bond, impacting cell activity.^140^ However, this strategy is particularly effective when coupled with protein-rich materials, which often contain aromatic amino acids—tryptophan, tyrosine, and phenylalanine—that can naturally interact with cyclodextrins.^141^ Indeed, cyclodextrins have been previously reported to interact with proteins, acting as artificial chaperones that can mediate and modulate protein folding and fibrillogenesis, and improve protein stability.^142–144^ Moreover, this mechanism allows the introduction of drugs and growth factors into the hydrogels, as excess cyclodextrins can bind other molecules containing hydrophobic groups. By combining this approach with ionic complexation using catechol-like moieties, this system has successfully produced matrices suitable for osteochondral regeneration.^145^

In a previous work by one of our groups, this strategy was applied on decellularized amniotic membrane, enabling the development of hydrogels that exhibit both tunable stress relaxation and strain-stiffening properties, while also preserving the native biochemical complexity of the ECM.^146^ Since this cross-linking strategy relies only on interactions between the cross-linker and aromatic amino acids within the protein matrix, it was observed that batch-to-batch differences in the mechanical properties were correlated to the aromatic amino acid content present in the matrix. Thus, this single parameter could be used to predict the mechanical properties of the material and, potentially, control them. These results demonstrate that the impact of the batch-to-batch variability can be mitigated by leveraging chemical strategies that target a single chemical moiety present in the material, thus coupling the mechanical properties of the scaffolds to the quantity of functional groups susceptible to chemical modification. The latter can be controlled by selectively pooling different batches.

### DNA Base Pairing

DNA represents yet another example of a naturally self-assembled and self-regulating nanostructure, which has served as an inspiration to the development of new scaffolds. The sequence-specific complementarity of nucleic acids enables the formation of highly stable and predictable nanostructures. As such, by incorporating nucleic acids as building blocks, it is possible to manipulate scaffolds at the nanoscale and generate complex, rationally designed nanostructures with high fidelity. Thus, DNA nanotechnologies have become burgeoning tools in the field of supramolecular chemistry.

A key example of the potential of DNA-based assemblies is DNA origami, in which short strands of DNA, known as staples, are designed to selectively fold a longer DNA strand (the template stand) to generate nanoparticles with predefined morphologies.^150^ Although these interactions occur at the nanoscale, the bottom-up assembly of such nanostructures enables the construction of scaffolds that span multiple length scales. Those include DNA nanotubes (Figure 5A), in which DNA strands are assembled into tiles—nanoscale modular units—that can further self-assemble into fibrillar structures extending to several micrometers in length (Figure 5B), emulating the architecture of ECM fibers.^147^

**Figure 5:**
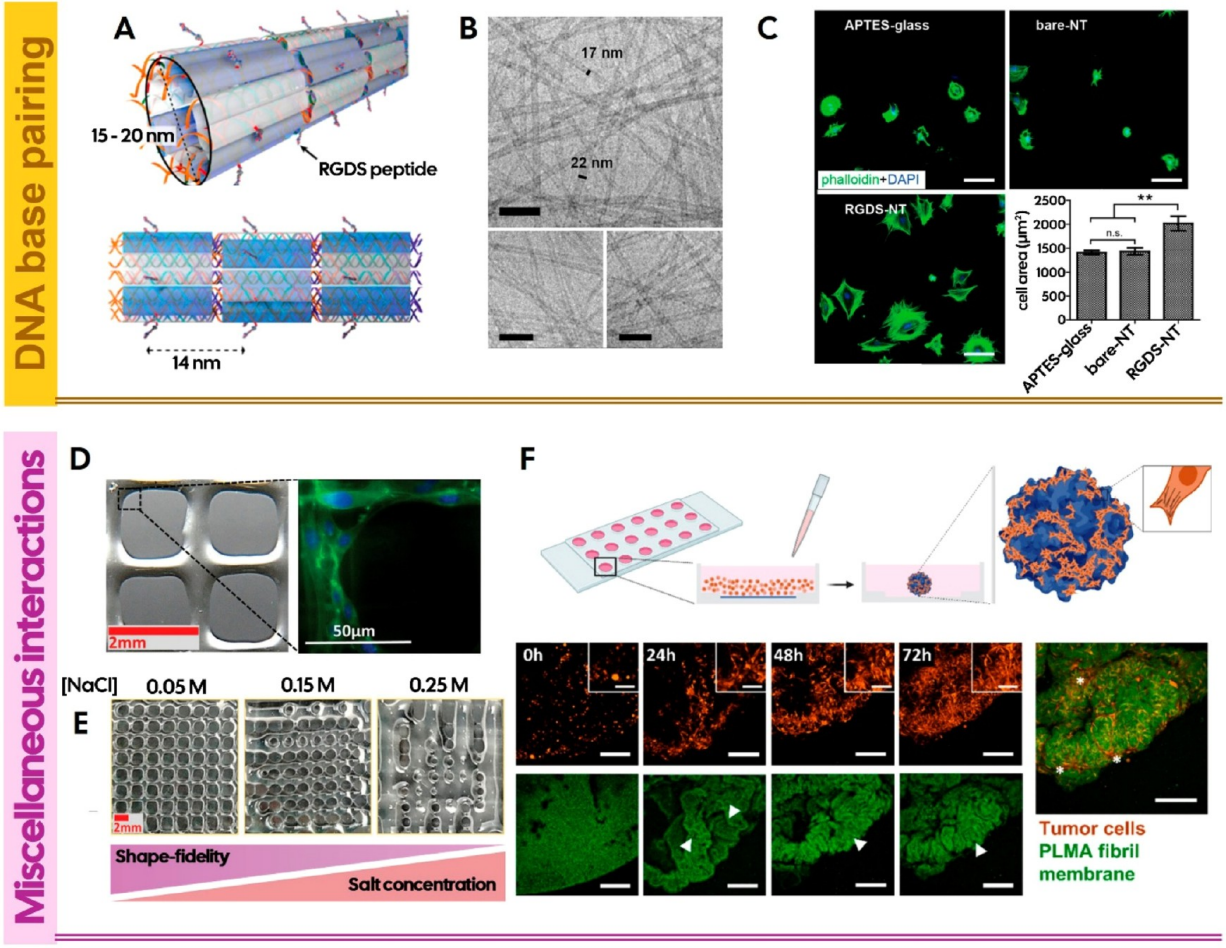
(A) RGD-functionalized DNA nanotubes (RGDS-NT) designed to self-assemble into fibrillar structures, extending several micrometers in length (B). Scale bar is 100 nm; (C) Fibroblasts adhered to all substrates, with greater spreading and a polygonal shape observed on the RGDS-NT-coated surface, along with actin bundling. Scale bar is 100 μm. Reproduced with permission from ref 147. Copyright 2015 American Chemical Society; (D) DAPI/phalloidin staining of U251 glioblastoma cells seeded on top of the printed HA-CHT coacervates after 7 days of culture. Reproduced with permission from ref 148. Copyright 2023 Wiley-VCH GmbH or related companies. All rights reserved, including rights for text and data mining and training of artificial intelligence technologies or similar technologies; (E) Pictures of the 3D-printed square mesh constructs with different NaCl concentrations (0.05 to 0.25 M) at pH 5, using low molecular weight HA (30–50 kDa) and chitosan (30 kDa). It was demonstrated that the ink performance can be tuned by adjusting parameters such as salt concentration, pH, and polyelectrolyte molecular weight. Notably, shape fidelity was significantly improved by lowering the salt concentration and increasing the pH from 5 to 6. Reproduced with permission from ref 148. Copyright 2023 Wiley-VCH GmbH or related companies. All rights reserved, including rights for text and data mining and training of artificial intelligence technologies or similar technologies; (F) Schematic representation of the human methacryloyl platelet lysate (PLMA) fibril-derived membrane and the traction forces applied by cells seeded on top, leading to the formation of membrane folds and eventual complete folding (top), and confocal microscopy images showcasing the development of cell/membrane aggregates over 3 days of culture (bottom). Scales bars are 250 (main images) and 100 μm (magnified images in the upper right corner and right image). Arrowheads and asterisks showcase the membrane folds forming during membrane folding and the cells stretched between the membrane folds, respectively. Reproduced with permission from ref 149. Copyright 2024 American Chemical Society.

While DNA-based artificial ECMs require functionalization with cell-recognition domains such as RGD to support cell-matrix interactions (Figure 5C), a key advantage of DNA-based structures over other synthetic or natural building blocks is that the introduction of these motifs can be precisely controlled. As such, it allows us to fine-tune the quantity, position, density and distance between the motifs in the final structure to maximize cell performance. Therefore, DNA-based constructs represent a highly promising strategy to generate highly tunable and complex scaffolds with high precision. However, this approach presents a major limitation, which is the prohibitive cost of DNA-based assemblies. To reduce costs, nucleic acids can be used not as building blocks but as tools to assemble other building blocks. One promising avenue involves the use of DNA chains as cross-linkers for biopolymers functionalized with DNA sequences. This approach has previously been used to engineer hydrogels with tunable stiffness and stress relaxation properties for cell and organoid culture.^151^

While the approaches described so far leverage the molecular recognition and complementarity between DNA base pairs, the versatility imparted by nucleic acid assemblies is not limited to these interactions. When mismatches of nucleobases occur and do not follow the usual Watson–Crick base pairing rules, other binding motifs, such as Hoogsteen base pairs, can be formed. In fact, Hoogsteen base pairs are able to create nondouble helix structures such as triplexes or quadruplexes.^152^ For example, in G-quadruplex structures, guanine (G) pairs with other G nucleobases instead of pairing with cytosine (C), creating a distinct configuration. The building blocks of G-quadruplexes are G-quartets (G4), formed by Hoogsteen-type hydrogen bonding interactions between four guanines in a square planar arrangement. Additionally, these structures are stabilized by the presence of cations, most commonly K^+^ ions, thus constituting another example of metal-ion coordination in natural systems. Through the π-π stacking of the G4 monomers, a columnar helical G-quadruplex structure is formed.^153–155^ Beyond their biological relevance, such as their role in telomere maintenance, DNA replication, transcription, and as targets for anticancer therapeutics,^156,157^ self-assembled G-quadruplex structures have garnered increasing attention as functional supramolecular motifs for the design of biomaterials. In particular, their ability to form multivalent interactions allows for the formation of entangled nanofibrillar networks that can retain a large amount of water leading to the formation of hydrogels with tunable mechanical and dynamic properties.^155,158–161^

### Miscellaneous Interactions

From a bioengineering perspective, cells are viewed as highly compartmentalized entities, divided into a multitude of specialized compartments known as organelles, each performing distinct functions that collectively govern cell survival and differentiation. Traditionally, this perspective has viewed organelles as delimited membrane-bound structures with well-defined boundaries, akin to the cell itself. However, recent findings have increasingly uncovered structures that result from the self-assembly of proteins and nucleic acids, which lack membrane boundaries yet still exert important roles in cell function. These membraneless organelles, also referred to as biomolecular condensates, arise from the coacervation and phase separation of molecules present in the cytoplasm.^162^ By examining the processes through which they are formed, it may be possible to uncover new mechanisms by which natural molecules self-assemble through supramolecular interactions.

Harnessing liquid–liquid phase separation to assemble biomolecules into new platforms for cell culture is not a novel concept. In fact, it is well-known that proteins and polypeptides assemble at the interface between liquid phases, generating stiff protein nanosheet structures.^163–165^ This strategy has already been successfully implemented to culture a wide variety of adherent cell types in water–oil and water–ionic liquid bioemulsions, demonstrating that the resulting interfacial nanostructures exhibit sufficient stiffness and structural integrity to support cell adhesion, proliferation and differentiation, while also inducing proper cell mechanotransduction.^166–172^

Another way to induce liquid–liquid phase separation is through tannic acid, a natural plant-derived polyphenolic compound with antibacterial, antiviral and anti-inflammatory properties that can establish hydrogen bonds with proteins and polysaccharides. Moreover, it also denotes the ability to bind different metal ions and to participate in π-π stacking interactions.^173^ Through its multivalent interactions with other (macro)­molecules, tannic acid promotes the formation of coacervates and hydrogels, an approach that has already been pursued to create strong adhesives,^174,175^ and smart materials.^176^

Naturally occurring polyelectrolytes, such as CHT and HA have been widely used in the design of advanced biomaterials and, more recently, in the development of coacervate-based inks with exceptional viscoelastic properties and printing fidelity, enabling both cell proliferation and biocompatibility (Figure 5D,E).^148^

However, the true versatility of coacervates extends far beyond 3D bioprinting. Owing to their cytomimetic properties, coacervates are also promising candidates for developing artificial cells. A striking example pertains to the use of coacervate microdroplets, formed by electrostatic interactions between quaternized (positively charged) and carboxymethyl (negatively charged) amylose to design a novel protocell model.^177^ The stabilization of these microdroplets through interfacial self-assembly of a triblock copolymer, consisting of hydrophilic, hydrophobic, and polyanionic motifs, is particularly noteworthy. With their application in both bioprinting and artificial cell design, coacervates are proving to be highly versatile, not only pushing the boundaries of biomaterials research, but also offering new opportunities to replicate the dynamic and biofunctional properties of the ECM for enhanced cell adhesion, proliferation, and tissue regeneration.

Oppositely charged natural polyelectrolytes, assembled via electrostatic interactions between them, have been also widely used to develop ECM-mimetic nanotopographic multilayered membranes to recapitulate the nanostructural and mechanical features of the native ECM. These multilayered membranes successfully replicate submicron topographical cues that direct cell alignment, elongation, and differentiation.^178^ Notably, such nanotopographical cues were sufficient to induce myogenic differentiation even in the absence of specific biochemical stimuli, underscoring the instructive role of physical ECM features. Polysaccharides stand out due to their structural resemblance to native ECM components, along with their intrinsic biocompatibility and biodegradability, making them ideal building blocks for enabling ECM-mimetic biomaterials. In fact, combining different natural polymers is a powerful way to unite the complementary properties of each one and to develop a wide repertoire of biomaterials of across multiple length scales.^179,180^

Returning to the processes that occur in our cells, it is also possible to identify novel mechanisms by which proteins and nucleic acids self-assemble, leading to liquid–liquid phase separation and the formation of membraneless organelles. Although the processes of protein dimerization and oligomerization have already been discussed in a previous section, there are other relevant processes that can be leveraged, such as the presence of intrinsically disordered regions in proteins, which promote protein–protein interactions.^162^ These processes can lead to the assembly of self-separating liquid phases, hydrogels, and insoluble aggregates, the latter of which are often associated with pathological conditions, such as prion diseases,^181^ or the deposition of amyloid fibrils.^182^ However, nontoxic amyloid structures have been identified and are increasingly being exploited as platforms for cell culture due to their similarities with the ECM.^183^ The standard procedure to generate amyloid fibrils for tissue engineering applications involves the acidification of a protein solution (generally to pH 2), followed by heating, typically to 80–90 °C.^184^ However, it is also possible to take advantage of other mechanisms for fibril formation, including concentration-dependent aggregation,^185^ or the use of chemical adjuvants that promote protein fibrillation. For example, one of our groups has pioneered the use of ionic liquids to promote the formation of amyloid fibrils from human platelet lysate.^149^ These fibrils were subsequently used to generate free-standing membranes which were incorporated into spheroids to achieve closer-to-native microtissue architecture (Figure 5F).

Although the interactions discussed in this section impart a wealth of dynamic mechanical properties to scaffolds, their exploitation remains a nascent field of research. Nevertheless, as these noncovalent assembly mechanisms become increasingly recognized as a fundamental component of a fully functional ECM, it is expected that this field will continue to flourish. However, it must be acknowledged that the relevance of noncovalent assembly would remain obscured, if not for the pioneering research developed by those working with synthetic supramolecular polymers. Indeed, while natural matrices remain the most popular option to create 3D cell culture platforms for disease modeling and drug validation, the most promising breakthroughs regarding the underlying mechanisms behind cell-matrix interactions have been uncovered in synthetic supramolecular matrices, which afford a greater degree of tunability and reproducibility. In the following section, we highlight these synthetic supramolecular platforms, and the extensive breakthroughs achieved to date in attempting to recreate the native ECM. While many synthetic polymers have been exploited in an attempt to develop artificial ECMs, including PEG,^131,186^ we put an emphasis on polymers that have attempted to emulate the noncovalent self-assembly processes found in the native ECM.

## A Supramolecular Toolkit to Recreate ECM Dynamics and Mechanical Properties

The complexity, variability and dynamicity of the ECM and other natural fibrillar networks are intrinsic features that allow them to adapt and respond to diverse stimuli and to cell activity, ultimately guiding cells toward the formation of different tissues. However, the same properties also pose a major challenge when attempting to use such natural systems as reliable models for studying how cells perceive and respond to specific biochemical and biomechanical cues. To overcome these limitations, scientists have turned to different classes of synthetic materials derived from peptides, small moleculesor inclusion complexes, which offer enhanced reproducibility and tunability, enabling the controlled emulation of individual ECM features. In fact, supramolecular assemblies have emerged as a promising class of next-generation biomaterials owing to their inherent adaptability, dynamic behavior, and precise control over structural, physicochemical and mechanical properties at the nanoscale. Approaches such as supramolecular copolymerization enable fine-tuning key parameters, including morphology, dynamics, and the spatial organization of bioactive ligands within these materials. Likewise the native ECM, these systems, which denote nanoscale structures determined by molecular recognition and self-assembly, can form complex and hierarchically organized macroscopic supramolecular architectures with customizable mechanical and biological functionalities. By leveraging these strategies, it has become possible to uncover underlying mechanisms of cell signaling and reproduce natural cell behaviors using scaffolds that retain only a fraction of the ECM’s complexity, while offering significantly greater design flexibility than natural matrices.^187,188^

### Elastin-Like Polypeptides

Elastin-like polypeptides (ELPs) are temperature-responsive biosynthetic polymers usually formed by the repetitive pentapeptide sequence -Val-Pro-Gly-X-Gly-. This sequence is derived from the hydrophobic domain of tropoelastin, the soluble precursor of elastin (an ECM protein, responsible for providing resilience and elasticity to the tissues), where X represents any amino acid except proline, as it would disrupt the peptide chain conformation.^189–191^ ELPs are highly modular, allowing precise tuning of the phase-transition temperature by varying either the chain length or the nature of the guest amino acid. Foundational studies on ELPs revealed that the increase in the hydrophobicity of the X residue leads to a decrease in the lower critical solution temperature (LCST), whereas hydrophilic substitutions result in an enhancement of the LCST.^192,193^ It is also proposed that the phase-transition phenomenon is driven by hydrophobic interactions, as the water molecules surrounding hydrophobic residues become less ordered upon heating above the LCST.^194^ Through this mechanism, ELPs undergo a conformational change from extended, hydrophilic chains into densely packed, hydrophobic coacervate aggregates that adopt a β-spiral architecture.^195,196^ ELP-based biomaterials have been widely used for drug delivery,^197–199^ and tissue regeneration.^195^ Illustrating the latter, ELPs were conjugated with acrylate moieties to create a photoresponsive 3D-printable bioink, in which the expression of specific ECM proteins was observed after only 18 days, showcasing the remarkable capacity of ELP-based biomaterials to recapitulate the native ECM.^200^ Furthermore, with the goal of obtaining higher ordered architectures, ELPs have been coupled to other synthetic polymers.^201,202^ For example, it has been shown that various morphologies, including polymersomes, interconnected worm-like micelles, and spherical micelles could be obtained by adjusting the hydrophilic ratio of the synthetic polymer in the diblock copolymer poly­(γ-benzyl-l-glutamate)-*b*-ELP (PBLG-*b*-ELP) (Figure 6A).^202^

**Figure 6:**
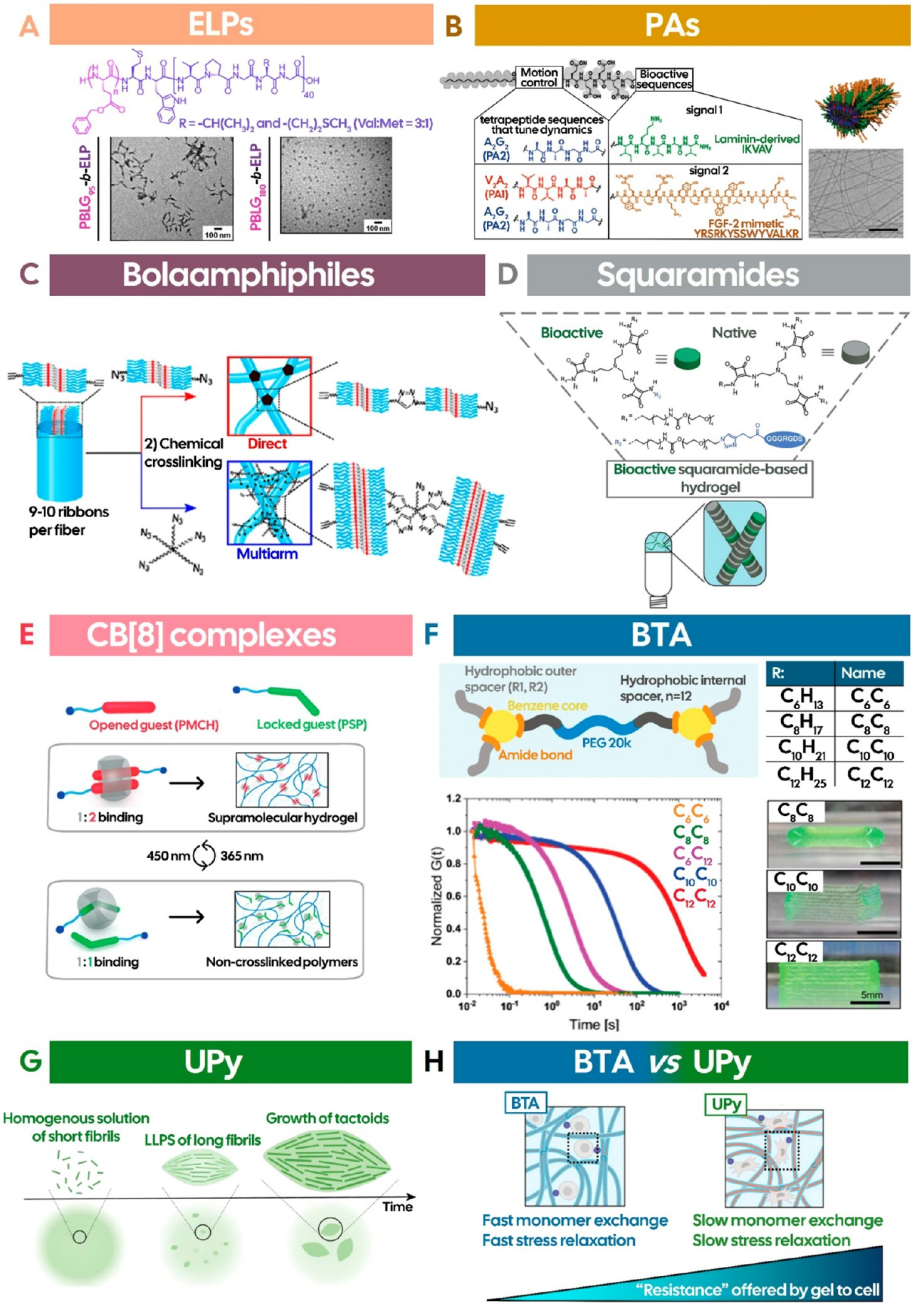
(A) Interconnected worm-like (bottom left) and spherical micelles (bottom right) formed through modulation of the hydrophilic ratio in the diblock copolymer poly­(γ-benzyl-l-glutamate)-*b*-ELP (top), highlighting its influence on the nanostructure morphology. Reproduced with permission from ref 202. Copyright 2017 The Royal Society of Chemistry; (B) PA functionalized with distinct bioactive motifs (IKVAV and FGF-2) and incorporating different tetrapeptide sequences (PA1 or PA2) to modulate the molecular dynamics can coassemble into high-aspect-ratio ribbon-like fibers, with enhanced molecular motion observed in the IKVAV–PA2 + FGF-2–PA1 coassembly, likely due to reduced intermolecular interactions arising from mismatched tetrapeptide sequences. Scale bar is 200 nm. Reproduced with permission from ref 205. Copyright 2021 The American Association for the Advancement of Science; (C) Schematic representation of triazole cross-linking in fiber-forming bisurea BA using azide- and alkyne-functionalized monomers (top) or a multiarm penta-azide linker (bottom). Reproduced with permission from ref 217. Copyright 2018 American Chemical Society; (D) Coassembly of native and RGD-functionalized SQ monomers drives the formation of bioactive supramolecular hydrogels. Reproduced with permission from ref 220. Copyright 2021 John Wiley and Sons; (E) Design strategy for the photoswitchable host–guest binding stoichiometries in supramolecular polymer networks. Reproduced with permission from ref 227. Copyright 2024 John Wiley and Sons; (F) Variation of the hydrophobic spacer length in BTA-PEG-BTA monomers (top) modulates the stress relaxation and the shape-fidelity of the resulting bioink (bottom). Longer hydrophobic spacer lengths (C_12_C_12_) show the slowest stress relaxation (bottom left) and the highest shape-fidelity (>80%, bottom right). Scale bars are 5 mm. Reproduced with permission from ref 235. Copyright 2023 Wiley-VCH GmbH or related companies. All rights reserved, including rights for text and data mining and training of artificial intelligence technologies or similar technologies; (G) Spontaneous liquid–liquid phase separation (LLPS) of UPy-Gly supramolecular polymers into tactoids driven by the continuous growth of fibrils. The process can be tuned with dextran as a macromolecular crowder, with higher concentrations accelerating LLPS and producing smaller and thinner tactoids. Reproduced with permission from ref 242. Available under a CC-BY 4.0. Copyright 2024 Springer Nature; (H) Illustration depicting the contrast in molecular (monomer exchange) and bulk (stress relaxation) dynamics between BTA- and UPy-based hydrogels. Reproduced with permission from ref 246. Copyright 2023 American Chemical Society.

### Peptide Amphiphiles

Peptide amphiphiles (PAs) are versatile self-assembling molecules composed of short peptide sequences covalently linked to hydrophobic alkyl tails. Their modular design allows precise control over molecular interactions: peptide domains near the hydrophobic tail are typically engineered to promote β-sheet formation, while residues farther from the alkyl tail are often charged to enhance the solubility in aqueous media and enable responsiveness to external stimuli, such as pH,^203^ and ionic strength.^204^ Moreover, PAs typically also contain bioactive epitopes for mediating targeted cellular responses.^205^ In aqueous environments, β-sheet formation combined with hydrophobic collapse of the alkyl tails drives the spontaneous organization of PAs into supramolecular 1D nanostructures, most commonly high-aspect-ratio cylindrical or ribbon-like fibers. These supramolecular nanostructures hold tremendous promise for biomedical applications, combining the dense, spatially controlled presentation of biological cues with the ability to guide cell behavior and influence signaling pathways. In addition, the PA-based nanostructures are also inherently biocompatible and biodegradable. Pioneering research has demonstrated that PAs can self-assemble into long nanofibers, forming ECM-mimetic scaffolds that direct cell functions.^206^ Since then, several studies have explored how PA molecular structure, particularly hydrophobic-tail length and peptide-sequence design control β-sheet formation, nanofiber morphology and stiffness, and ultimately biological function.^207–209^ However, while significant progress had been made in controlling the molecular features underlying PAs self-assembly,^210^ it became clear that the nanostructural organization alone could not fully recapitulate the instructive functions of the ECM.

To address this gap, the scientific community made important contributions by pushing the boundaries toward the development of multicomponent PA-based supramolecular hydrogels for regenerative medicine applications.^205,211–214^ In particular, a landmark study reported the development of a PA-based hydrogel through the self-assembly of specific PA sequences, integrating two distinct biological signals: a laminin-derived Ile-Lys-Val-Ala-Val (IKVAV) motif to stimulate neural stem cell differentiation into neurons, and a fibroblast growth factor 2 (FGF-2) mimetic peptide (YRSRKYSSWYVALKR) to promote cell viability and proliferation (Figure 6B).^205^ Moreover, by varying nonbioactive regions of the peptide sequence, the molecular motion within the nanofibrillar network could be modulated, demonstrating that the interplay between dynamic molecular behavior (i.e., greater supramolecular motion) and biological signaling critically enhances spinal cord regeneration *in vivo*.

### Bolaamphiphiles

Although significant progress has been made in designing synthetic supramolecular materials that mimic the nanostructural and biochemical complexity of the ECM, reproducing its unique nonlinear elasticity, known as strain-stiffening, without sacrificing reversibility remains a key challenge. As such, scientists have increasingly focused on modular monomeric building blocks, such as bolaamphiphiles (BA). These amphiphilic molecules, defined by a hydrophobic core flanked by two hydrophilic end groups, self-assemble in water into various well-defined supramolecular structures. For instance, when decorated with complementary bisurea motifs and oligo­(ethylene glycol) (OEG) chains, the BA can form rodlike micelles through the aggregation of 9–10 ribbons into semiflexible fibers via intermolecular urea–urea hydrogen bonds and hydrophobic interactions. Their mechanical properties can be precisely controlled by varying the length of the PEG chains or the size of the hydrophobic spacer.^215,216^ Additionally, azide- and alkyne-modified bis-urea BA can be incorporated to enable direct triazole cross-links between the self-assembled fibers, or a multiarm penta-azide linker can be used to facilitate cross-linking between and within fibers (Figure 6C).^217^ This hybrid supramolecular-covalent approach enables the development of hydrogels composed of self-assembled, semiflexible BA-derived nanofibers, which exhibit self-healing properties and strain-stiffening behavior assigned to their soft bending characteristics in response to stress,^217,218^ closely mimicking the mechanical properties of biological tissues. When compared to fully supramolecular systems, these reinforced networks can withstand greater stress and offer enhanced control over the mechanical properties through modular chemical design. Beyond their mechanical versatility, supramolecular assemblies featuring bisurea motifs can be functionalized with ECM-derived peptide sequences to form microfibrous rafts that promote human induced pluripotent stem cells adhesion and sustain their pluripotency, while avoiding the undesired clumping observed with conventional microcarrier beads.^219^

### Squaramides

As ditopic hydrogen bonding units with two N–H donors and two C=O acceptors on a cyclobutenedione ring, squaramides (SQ) enable directional hydrogen bonding, leading to precise supramolecular organization into well-defined nanostructures. Similarly to bisurea BA, the influence of hydrophilic and hydrophobic segment lengths on the structure-morphology relationship has also been investigated in SQ-based BA.^216^ It has been demonstrated that a hydrophobic chain length of 8 carbons is required to support the formation of fibrillar aggregates when combined with a fixed OEG chain (*m* = 11). In contrast, increasing the PEG chain up to *m* = 36, while keeping the hydrophobic spacer constant at 10 carbons, led to a shift from fibrillar to spherical morphologies, attributed to the growing steric hindrance of the longer PEG chains. By changing the molecular design of SQ from a linear structure to a tripodal monomer—in which three SQ units are incorporated within a hydrophobic core composed of tris­(2-aminoethyl)­amine, aliphatic chains, and peripheral hydrophilic domains—a supramolecular hydrogel has been developed through the coassembly of native and RGD-functionalized SQ monomers (Figure 6D), creating a system that functions as an alternative 3D matrix to Matrigel for HepG2 differentiation. Moreover, it illustrates how the coassembly of bioactive and nonfunctional monomers triggers the formation of multifunctional hydrogels that better mimic the ECM while requiring minimal amounts of the bioactive component.^220^

### Cucurbit­[*n*]­urils

Cucurbit­[*n*]­urils (CB­[*n*]­s, *n* = 5–8, 10, 13–15) have emerged since the early 2000s as a distinctive class of supramolecular compounds, demonstrating highly specific host–guest recognition and pronounced stimuli-responsiveness, opening new avenues for the design of dynamic and adaptive materials.^221–223^ Notably, CB[7], with its larger cavity compared to CB[6] and CB[5]—capable of binding both aliphatic and aromatic groups—has received considerable attention within the biomaterials field. In particular, the development of CB[7]-Phalloidin derivatives are used for the fluorescent labeling of F-actin^224^ in fixed cells and CB[7]-peptide conjugates for receptor-mediated cellular uptake.^225^ Extending this host–guest chemistry even further, CB[8] offers an additional level of structural sophistication. With its larger, barrel-shaped cavity, CB[8] stands out for its ability to accommodate two guest molecules simultaneously, forming a stable 1:1:1 ternary complex.^223^ This distinctive inclusion complex opens new avenues for the development of dynamic and reversible supramolecular hydrogels, such as those based on CB[8]-PA and CB[8]-spiropyran complexation.^226,227^ Illustrating the latter, scientists have used a spiropyran derivative to fit CB[8]’s cavity, enabling the creation of light-responsive hydrogels in which the host–guest interactions depend on the light-induced isomerization of the guest (Figure 6E). Under blue light, the spiropyran remains in a ring-closed nonplanar form (PSP), forming a 1:1 complex that does not allow cross-linking, whereas UV light triggers the isomerization of spiropyran to a ring-opened planar protonated merocyanine form (PMCH) which engages in a ternary complex, resulting in a cross-linked network. This reversible isomerization mechanism allows the hydrogels to rapidly alternate between soft and stiff states under light irradiation, significantly boosting their self-healing and shape-remodeling performance.^227^ Beyond spiropyrans, CB[8] has also been used to form homoternary complexes with azobenzene derivatives.^228–230^

### Benzene-1,3,5-tricarboxamide

Discotic molecules with C_3_-symmetry, particularly those based on benzene-1,3,5-tricarboxamide (BTA) cores, have been extensively studied as monomers for supramolecular polymerization. In aqueous environments, the functionalization of BTA molecules with hydrophobic C_12_ spacers and tetra­(ethylene glycol) (EG_4_) side chains have shown to promote the formation of multimicron, 1D supramolecular fibers. These polymers are stabilized through a synergistic interplay of π–π stacking between the aromatic cores, 3-fold hydrogen bonding via the amide groups, and hydrophobic interactions, forming chiral double helical polymers.^231^ Varying the length of the hydrophobic spacer influences the extent to which the hydrogen bonding motifs are exposed to water, with a C_12_ spacer being optimal to induce a stable assembly by creating sufficiently large hydrophobic pockets around the BTA core to shield the amide–amide intermolecular hydrogen bonds from the surrounding water. The dynamic nature of BTA-based assemblies, characterized by the homogeneous exchange of monomers along the fiber length,^232,233^ closely mirrors the adaptative and constantly remodeling behavior of the ECM, turning them into a promising platform for the development of ECM-mimetic materials. Recently, biomimetic BTA-based hydrogels with tunable stiffness and dynamics have been developed by combining conventional EG_4_-BTA with a telechelic BTA-PEG-BTA monomer, in which two BTA units are connected by a 20 kDa PEG chain. Notably, adjusting the ratio of the two monomers allowed the modulation of the mechanical properties and network dynamics, which in turn influenced cell aggregation within the hydrogel.^234^ Using the telechelic BTA-PEG-BTA monomer, variations in molecular design, particularly in the hydrophobic spacer length (Figure 6F, top) enable fine-tuning key rheological properties such as viscoelasticity and stress relaxation (Figure 6F, bottom), both of which are essential for engineering ECM-like bioinks.^235^

Electrostatic interactions between positively charged macromolecules and negatively charged cell membrane components, such as proteoglycans, play a key role in cellular recognition and signaling. Leveraging this principle, supramolecular BTA fibers—assembled from neutral and cationic BTA monomers—are capable of binding to cell membranes via charge-mediated endocytosis. This process enables intracellular delivery of hydrophobic guest molecules and negatively charged short interfering RNA sequences, previously encapsulated in the core and on the periphery of the BTA fibers, respectively. This design offers a versatile platform for delivering molecules that typically exhibit poor membrane permeability and are prone to degradation.^236^ Beyond intracellular delivery, BTA fibers bearing biomolecular moieties, such as benzoxaborole, can anchor to human red blood cells through dynamic covalent bonds with sialic acid residues present on their membranes.^237^ Moreover, the incorporation of RGD cell adhesion motifs into BTA fibers enables the formation of supramolecular hydrogels that promote enhanced cell spreading.^238^

### Ureido-Pyrimidinones

While BTA-based supramolecular assemblies, with their fast monomer exchange, are well-suited for mimicking the adaptive behavior of the ECM, such dynamic behavior can compromise their effectiveness in applications that demand more stable, less dynamic, and long-lasting structures. To overcome these challenges, supramolecular systems based on ureido-pyrimidinone (UPy) building blocks have garnered increasing attention. UPy molecules form dimers through self-complementary quadruple hydrogen bonding in a donor–donor–acceptor–acceptor configuration, with additional urea or urethane groups enabling lateral stacking. To promote the assembly of elongated nanostructures in aqueous media, hydrophobic spacers are attached to the urea groups to shield the hydrogen bonding motifs from water, while OEG or PEG side chains are incorporated to provide water solubility, enabling the formation of more robust fibers. Whitin the extensive library of studied UPy monomers, two are particularly crucial for the development of biomaterials: the more robust monofunctional (M-type) UPy, comprising a peripheral glycinamide group, and the dynamic bifunctional (B-type) UPy, in which PEG chains are telechelically modified with UPy motifs (UPy-PEG-UPy). The B-type monomer self-assembles into fibers capable of cross-linking, resulting in transient supramolecular hydrogels that exhibit promising life-like properties such as adaptability, self-healing, and pH-responsiveness, making them compelling candidates for intrarenal or myocardial drug delivery.^239–241^ Despite its inability to form hydrogels on its own, the M-type UPy monomer has demonstrated the ability to generate highly anisotropic aqueous liquid droplets, known as tactoids, through liquid–liquid phase separation driven by the continuous growth of robust supramolecular polymers (Figure 6G).^242^

Although the incorporation of RGD adhesion motifs is widely employed to promote cell spreading in UPy-based supramolecular hydrogels,^243–245^ there is a growing interest in understanding the role of the network dynamics in regulating bioactivity. By coassembling slow- (M-type) and fast-exchanging (B-type) UPy monomers, transient hydrogel networks with tunable dynamics can be engineered. Interestingly, in excessively dynamic networks, cell-adhesive UPy monomers (UPy–cRGD) become ineffective due to rapid binding/unbinding kinetics, which hinder stable ligand engagement and fail to retain bioactive motifs within the supramolecular fibers, ultimately impairing the formation of adhesion sites via integrin–cRGD binding. Therefore, dampening the exchange dynamics by increasing the M-type content relative to the B-type is crucial to restore cell adhesion.^243^ Furthermore, the precise modulation of the effective ligand concentration (cRGD) within UPy fibers provides a powerful approach to regulate epithelial cell polarity and adhesion.^244^ Additional biochemical complexity can be introduced into UPy-based hydrogels by incorporating UPy-functionalized collagen type I-mimicking peptides. This, together with the increased bulk stiffness, leads to greater nuclear translocation of yes-associated protein (YAP)—a well-known marker of mechanotransduction—compared to dECM hydrogels, highlighting that minimal biochemical cues combined with appropriate mechanical properties are sufficient to induce mechanotransduction.^188^

### Contrasting Supramolecular Dynamics in UPy- and BTA-Based Assemblies and Synergistic Effects

Importantly, when M- and B-type monomers are coassembled into three-component UPy- or BTA-based hydrogels—combining monofunctional, bifunctional, and RGD-functionalized building blocks—the choice of the supramolecular motif (BTA vs UPy) dictates the monomer exchange kinetics and, therefore, the material’s bulk behavior.^246^ More specifically, BTA monomers exchange roughly three times faster than UPy, leading to faster stress relaxation in the resulting hydrogel, which prevents the cells from generating the necessary tension for effective spreading. By contrast, UPy-based hydrogels sacrifice some dynamic adaptability for slower monomer exchange and greater mechanical resistance, which withstand cell traction forces (Figure 6H). Thus, the contrast between BTA and UPy systems highlights how molecular dynamics can be precisely tuned for optimal cell-matrix interactions.

To better mimic the (non)­linear elasticity of the ECM, recent efforts have focused on the coassembly of UPy and BTA supramolecular monomers—individually or in combination—with strain-stiffening synthetic covalent polymers such as polyisocyanide (PIC). PIC is one of the few synthetic polymers that exhibit strain-stiffening behavior, a phenomenon commonly observed in natural fibrous proteins, such as collagen. In fact, the PIC+UPy+BTA networks produced multicomponent fully synthetic hydrogels that synergistically combined and amplified the unique contribution of each individual component to achieve properties closer to those of the native ECM—UPy fibers imparted increased linear stiffness, BTA fibers extended the stiffening regime, and the overall network exhibited a stress relaxation profile comparable to that of the single-component PIC gel. Furthermore, the incorporation of cRGD motifs into the supramolecular fibers—particularly into the robust UPy fibers—enhanced the bioactivity of the three-component dynamic hydrogels, as demonstrated by their intrinsic ability to support human normal dermal fibroblast cell spreading.^247^

The ECM constitutes an extremely complex biochemical milieu that encompasses a wide variety of bioactive cell signaling molecules and moieties that are essential for enabling proper cell-matrix interaction. While synthetic supramolecular materials and systems can be engineered to replicate individual features of the native ECM, designing an artificial matrix that fully recreates its multifaceted nature remains an insurmountable challenge—likely requiring the creation of a material as complex as the ECM itself. Despite the persistent issue of batch-to-batch variability in natural-derived scaffolds, which also serve as a reminder that such variability is inherent to the ECM, these materials continue to play a major role in artificial ECM development owing to their intrinsic compositional resemblance with the ECM components. This raises a fundamental question: rather than striving to completely replace one approach with another, could hybrid materials—uniting the control, dynamicity and tunability afforded by synthetic supramolecular systems with the intrinsic biofunctionality of natural polymers—pave the way toward truly biomimetic artificial ECMs?

In the following section, we focus on those systems that aim to combine the efforts of separate molecules to achieve something greater than the sum of its parts by merging natural and synthetic supramolecular polymers into a single system with augmented properties.

## Toward Functional Diversity: Hybrid Materials

The combination of different natural polymers has already proven to be an effective strategy for designing materials with improved biofunctionality. For example, by blending ALG with HA, an abundant ECM component, and gelatin, which enhances bioactivity and promotes cellular interactions, it is possible to formulate a bioink that supports long-term cell viability, promotes cell spreading and elongation, and enables dynamic cell–matrix interactions consistent with native ECM behavior.^248^ This demonstrates how natural components can be synergistically combined to enhance biological performance, while avoiding extensive chemical modification. However, their limited stiffness, dictated by a specific concentration window, along with their limited processability and dynamicity, restrict their use in more demanding applications. Additionally, their physicochemical properties are challenging to modulate with precision, limiting their versatility in the rational design of tailored biomaterials. In contrast, synthetic (supramolecular) polymers offer structural versatility and tunable physicochemical and mechanical properties, yet lack the biological cues essential for promoting cell–material interaction.^249^ As such, the integration of natural and synthetic (supramolecular) polymers through dynamic noncovalent self-assembly is an emerging area that aims to merge biology with chemistry, enabling the development of hybrid materials that better mimic the multifaceted nature of the native ECM, where the bioactivity of natural components is complemented by the tunability of synthetic (supramolecular) matrices (Figure 7).

**Figure 7:**
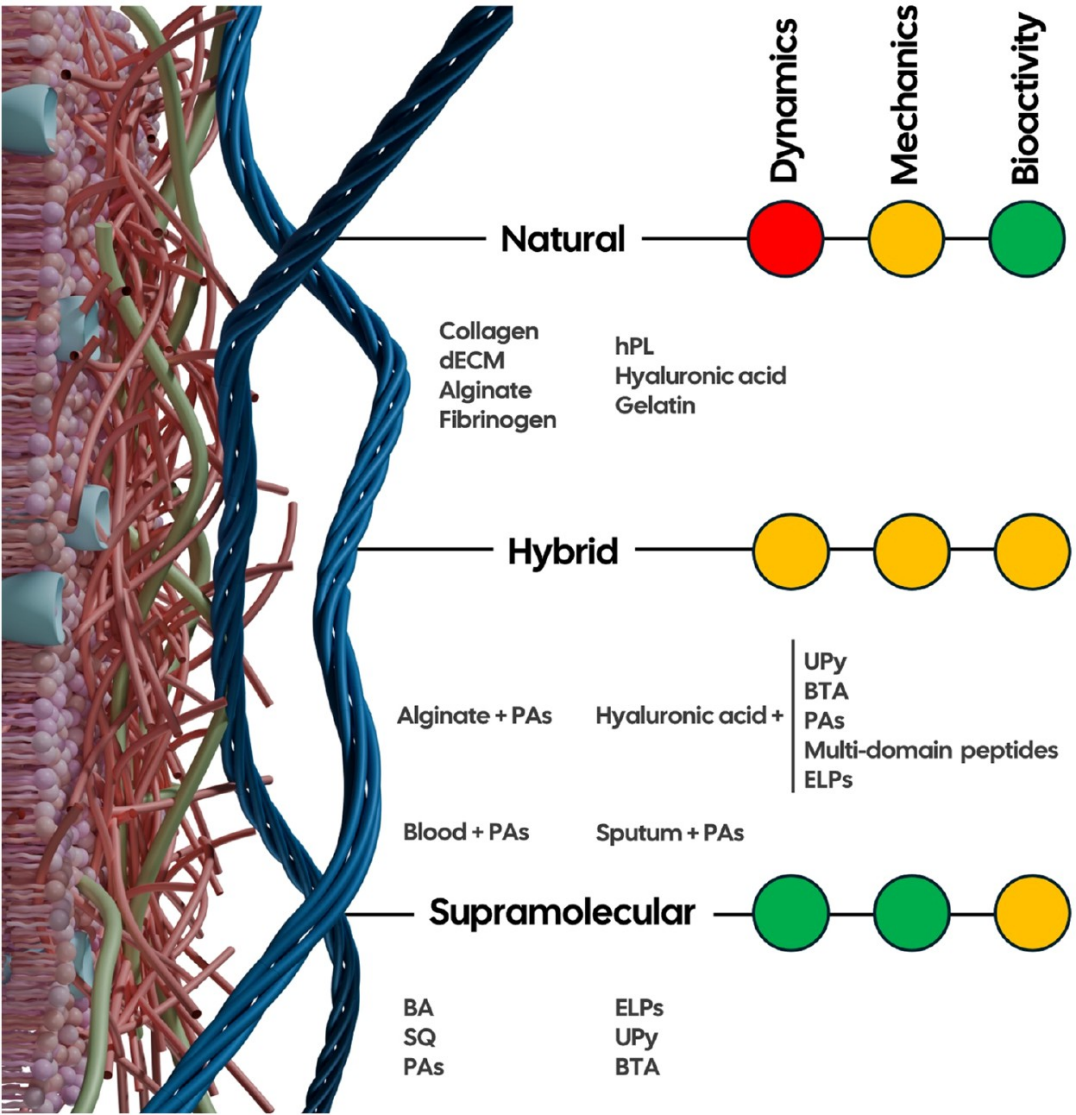
Landscape of natural, synthetic (supramolecular), and hybrid polymers toward ECM-mimetic platforms, with a color scale indicating the level of resemblance to key ECM features—dynamics, mechanics, and bioactivity. Green represents high similarity, yellow moderate, and red low similarity. Figure created using Blender software (4.3.2). Adapted with permission from ref 218. Copyright 2024 American Chemical Society.

Among the hybrid strategies, ALG stands out for its versatility in forming both bulk hydrogels with tunable mechanical properties,^250^ and nanostructured films via electrostatic interactions with cationic PAs.^251^ Beyond serving as a matrix component, the anionic nature of HA can also enable the coassembly with positively charged PAs, forming multilayered nanofilms,^252^ hydrogels,^253^ or macroscopic sacs^254^ through electrostatic complexation. Furthermore, when combined with multidomain peptides bearing distinct biochemical motifs—including mineralizing, integrin-binding, and osteogenic sequences—HA contributes to the formation of nanostructured interfacial membranes that simultaneously offer biomimetic structural organization and biochemical functionality.^255^ In addition, the incorporation of HA into dynamic, fiber-forming BTA-based^256^ or UPy-based^188^ synthetic supramolecular systems enables the design of hybrid matrices with nanoscale precision that recapitulate both the fibrillar architecture and bioactivity of native ECM.

Another interesting class of ECM-mimetic materials leverages natural polysaccharide-*b*-ELP block copolymers. By conjugating polysaccharides, such as dextran and HA to ELPs, scientists have enabled the self-assembly of nanoparticles through temperature-triggered processes, providing a promising platform for designing smart and bioactive nanocarriers for biomedical applications.^257^ Nonetheless, it is important to acknowledge that the integration of natural and synthetic polymers also presents inherent challenges. Despite their complementary properties, these materials can require distinct processing conditions, including differences in solvent compatibility, temperature sensitivity, and concentration ranges, which must be carefully harmonized. As such, the development of hybrid materials is not merely a matter of mixing two systems, but rather a process of optimization to ensure their synergistic functional combination.

Although much attention in ECM-inspired materials has been devoted to reproducing its fibrillar nanoarchitecture, the native ECM is much more than just a fibrous scaffold. It is a dynamic reservoir of growth factors and bioactive molecules which plays a crucial role in regulating cellular behavior. In this context, more complex biological sources, such as whole blood or specific media formulations, have emerged as unconventional but powerful building blocks for the assembly of hybrid materials. For example, the coassembly of PAs with human blood during coagulation leads to the formation of hybrid, clot-like hydrogels which accurately mimic the key biochemical and structural features of the regenerative hematoma, with a continuous supply of growth factors.^258^ In another application, the same supramolecular building blocks have been combined with artificial sputum medium to engineer living materials that can form functional 3D biofilms, which can be used to study antibiotic responses.^259^ Overall, the integration of biologically complex fluids as active components represents a shift toward next-generation hybrid systems that not only provide structural support but also confer biochemical functionality, more faithfully emulating the multifaceted nature of the native ECM.

## Conclusions and Future Perspectives

The ECM that sustains our tissues is an exceptional scaffold, one that continues to elude our attempts to reconstruct it. Inspired by the complex nanostructure of our tissues, both at the mechanical and the biochemical level, as well as the composition and the intricate dynamic behavior, scientists have been pursuing diverse approaches toward rebuilding and mimicking the native ECM in various ways. This has turned out to be a *tour de force*.

In this Perspective, we have described the recent progress in the development of artificial matrices to better recreate the native ECM. Several examples, ranging from either fully natural or synthetic matrices to hybrid systems have been described, while pointing out their main challenges and opportunities. Natural-based polymers are imparted with bioactivity, but lack control over the physicochemical and mechanical properties, dynamic behavior, and reproducibility. Supramolecular polymers offer structural versatility, tunable physicochemical and mechanical properties, and modular dynamics, yet lack biological cues to promote cell-material interactions. As such, although the developed materials are still simple when compared to the native ECM, exciting breakthroughs have already been accomplished.

The field has made considerable progress, as the introduction of chemical processing and supramolecular assembly has been leveraged to mitigate the inherent challenges of Nature-derived matrices, such as their limited mechanical properties and batch-to-batch variability. At the same time, biological moieties have increasingly been incorporated into synthetic supramolecular systems to overcome their biochemical inertness and drive cell fate. These approaches have blurred the boundaries between natural and synthetic supramolecular systems, causing them to converge. This has resulted in the development of advanced hybrid systems, denoting emerging properties and nanostructural features, which cells can sense and respond to by modulating their functions. In fact, the synergistic interplay between natural macromolecules and small synthetic building blocks that self-assemble into supramolecular polymers at the nanoscale is at the forefront in enabling more complex and larger biofunctional matrices that could better recreate the native ECM’s complexity, dynamics, bioactivity and mechanical signals. Indeed, the recent advances in the development of hybrid systems and the possibility of inserting protein fragments to add biofunctionality have increased our fundamental understanding of cell-material reciprocal interactions and cell–cell interactions. Despite the progress, the field is still in its infancy, as clearly shown by the few examples and current moderate performance of hybrid systems, illustrated in Figure 7.

One exciting field that should be highlighted is *de novo* protein synthesis, the result of decades of research in the field of protein engineering, allowing us to synthesize molecules that do not yet exist in Nature. Recent advances in computational approaches provide a new framework to advance even further and accelerate the process of *de novo* protein design to craft next-generation molecules which exhibit predefined composition, 3D structure and biomechanical properties, and dynamic programmable functions, which has been awarded with the Nobel Prize in Chemistry in 2024.^260^

Another area which is emerging and challenging the *status quo* is the development of minimalist approaches by resorting to low-material amounts or even scaffold-free structures aiming to put the focus on the assembly of size- and shape-tunable single or multicellular architectures denoting high cellular density. Those include cell fixed structures (*e.g.*, cell-only spheroids and fibers,^261,262^ or extracellular vesicles^263^), cells functionalized with photoswitchable proteins,^264,265^ or even living materials harnessing the self-assembly between cells, offering promising opportunities in the bottom-up assembly of more complex and programmable human tissue-like structures, from cellular building blocks, resembling native tissues.^266^ By applying the principles of materials’ design to cells themselves, this research demonstrates the possibility to merge biology and chemistry, breaking down the boundaries between the two fields and paving the way toward the artificial reconstruction of life itself.

All in all, while a fully functional artificial ECM may yet remain out of our grasp, the individual achievements of those working with either natural matrices or synthetic (supramolecular) polymers, as well as the ample opportunities we have identified for cooperation bring hope that the continued dialogue and synergistic interplay between the natural and synthetic worlds will be key to driving exciting breakthroughs in the development of complex bioinspired materials, with combined properties that cannot be obtained by the individual components, to better emulate the complexity and dynamics of living systems.

The question here is how far can we push this complexity? And which factors, such as mechanics, bioactivity, and dynamics, are most important? We believe that the synergy of all factors is crucial, and we are committed to continuing joining efforts from our groups, together with the scientific community at large, in moving forward toward more complex and dynamic materials and systems, pursuing the dream of creating a fully functional artificial ECM in the decades to come.
